# The cytocompatibility of graphene oxide as a platform to enhance the effectiveness and safety of silver nanoparticles through in vitro studies

**DOI:** 10.1007/s11356-023-30151-1

**Published:** 2023-10-12

**Authors:** Barbara Strojny-Cieślak, Sławomir Jaworski, Mateusz Wierzbicki, Michał Pruchniewski, Malwina Sosnowska-Ławnicka, Jarosław Szczepaniak, Agata Lange, Piotr Koczoń, Marlena Zielińska-Górska, Ewa Sawosz Chwalibóg

**Affiliations:** 1 Department of Nanobiotechnology, Institute of Biology, Warsaw University of Life Sciences, Warsaw, Poland; 2 Department of Chemistry, Institute of Food Sciences, Warsaw University of Life Sciences, Warsaw, Poland

**Keywords:** Graphene oxide, Ag nanoparticles, Biocompatible platform

## Abstract

The increasing emergence of antibiotic-resistant bacteria and the need to reduce the use of antibiotics call for the development of safe alternatives**,** such as silver nanoparticles. However, their potential cytotoxic effect needs to be addressed. Graphene oxide provides a large platform that can increase the effectiveness and safety of silver nanoparticles. Graphene oxide and silver nanoparticles complex applied as a part of an innovative material might have direct contact with human tissues, such as skin, or might be inhaled from aerosol or exfoliated pieces of the complex. Thereby, the safety of the prepared complex has to be evaluated carefully, employing a range of methods. We demonstrated the high cytocompatibility of graphene oxide and the graphene oxide–silver nanoparticles complex toward human cell lines, fetal foreskin fibroblasts (HFFF2), and lung epithelial cells (A549). The supporting platform of graphene oxide also neutralized the slight toxicity of bare silver nanoparticles. Finally, in studies on *Staphylococcus aureus* and *Pseudomonas aeruginosa*, the number of bacteria reduction was observed after incubation with silver nanoparticles and the graphene oxide–silver nanoparticles complex. Our findings confirm the possibility of employing a graphene oxide–silver nanoparticles complex as a safe agent with reduced silver nanoparticles’ cytotoxicity and antibacterial properties.

## Background

Graphene is a one-atom-thick, two-dimensional carbonaceous material. Its planar structure is composed of sp^2^ hybrid carbon atoms, where every atom is connected by three δ bonds to the adjacent carbon atom, forming a honeycomb lattice crystal structure (Geim and Novoselov 2007).The electron cloud on the surface makes graphene an excellent conductor; thus, the studies on innovative graphene-based electronics were the first to develop rapidly (Basu et al. 2010; Wang et al. 2017; Yildiz et al. 2021). Later, the biomedical, food industrial, and ecological fields started to develop an interest in applying graphene, usually in the form of a nanocomposite (Bacakova et al. 2020; Garavand et al. 2020). With the development of fabrication and synthesis methods, numerous possibilities for functional modifications have arisen, which is especially important for the potential biological and medical applications. Pristine graphene is a hydrophobic material, which causes agglomeration in water-based media; thus, it might be problematic for in vivo applications. The oxidized form of graphene, called graphene oxide (GO), overcomes this problem (Jaworski et al. 2021). The introduction of oxygen-rich groups, such as carbonyl, hydroxyl, epoxy, or carboxy groups, improves the material’s solubility in an aqueous environment and stabilizes the flakes because the groups push away the single layers of GO and widen the gap between them (Gao et al. 2019; Mouhat et al. 2020). The groups may also serve as anchors for further functionalization. The most important consequence of increased hydrophilicity is the acquired biocompatibility of GO in comparison to pristine graphene. GO can be produced from graphite by several cost-effective methods, such as Hummers’, Marcano’s, and others; thus, it is cheaper than pristine graphene (Marcano et al. 2010; Liao et al. 2018). The choice of the initial material and synthesis method results in slightly varied GO flakes, which may differ in size and oxygen-rich groups’ proportion and distribution, resulting in significant or subtle differences in physicochemical properties and subsequent biological activities (Strojny et al. 2020).

The active surface of GO can be non-covalently modified with simple methods like mixing or self-assembly or by more sophisticated methods based on chemical reactions, leading to covalent modifications (Yu et al. 2020). This makes GO a promising platform for the delivery of active compounds like drugs, ions, proteins, or even nanoparticles. Applying GO as a supporting platform ensures even distribution of the agent and, by preventing unwanted agglomeration or aggregation, increases its stability (Parnianchi et al. 2018; Yu et al. 2020). Among the active compounds that can be applied to GO flakes or sheets are metal nanoparticles, such as gold, copper, titanium dioxide, or silver nanoparticles. Silver nanoparticles (Ag) have an outstanding range of activity and possible applications (Bold et al. 2022; Bhakya et al. 2016); however, the main problem is the aggregation of the particles. Thus, the incorporation of Ag onto GO might increase their stability, but it is also possible to obtain novel or synergistic effects of such a complex (He et al. 2018). Ag have unique optical and catalytic properties; thus, they might be applied in innovative electronic devices or sensors (Lorca-Ponce et al. 2023). Another significant feature is their strong antibacterial activity (Bruna et al. 2021; Wierzbicki et al. 2019); therefore, nanocomplexes of GO and Ag might serve as antimicrobial agents, additives, or compounds in novel wound dressings with prolonged activity (Azizi-Lalabadi et al. 2020; Mariadoss et al. 2020).

A GO:Ag complex could be applied as an antimicrobial agent on solid surfaces, be added to liquid disinfectants or medical fabrics, or serve as a compound for the aforementioned wound dressings, as it has been presented in our recent research (Lange et al. 2022). However, in each of these cases, either the material has direct contact with human tissues, such as skin and open wound tissue, or there is a high probability of indirect contact by touch or inhalation of either aerosol or exfoliated pieces of the complex. Therefore, it is extremely important to study the possible effects on humans and determine how such a complex and its primal components, GO and Ag, affect living cells. The cytotoxicity and biocompatibility of nanomaterials and nanoparticles are challenging because nanostructures sometimes tend to interact with standard, widely used test compounds (Strojny et al. 2020), which can be caused by interference with electron transport, component binding, or unique optical properties. It is crucial to apply different methods, study each of the newly composed materials separately, and carefully interpret the results. Confirmation of the biocompatibility of GO and Ag might not only facilitate their introduction as an innovative antimicrobial agent but also promote studies of the new possibilities for further applications of a GO:Ag complex in tissue engineering.

In the presented work, we propose an elegant method for obtaining a GO:Ag complex, without the use of harsh chemicals, with no need for purification, and minimizing the risk of contamination of the complex and possible toxicity during application. The potential cytotoxicity of the GO:Ag complex and its initial compounds, GO and Ag, was studied on two different human cell lines, fetal foreskin fibroblasts (HFFF2) and lung epithelial cells (A549). To obtain the most objective results, we employed several methods to determine cell viability, membrane integrity, proliferation rate, oxidative stress, apoptosis induction, and cell morphology. Additionally, we present an example of the antibacterial properties of the complex using two bacterial strains, *Staphylococcus aureus* and *Pseudomonas aeruginosa.*

## Material and methods

### Nanomaterials

GO was obtained from Nanopoz (Poznan, Poland) in the form of a 4000 mg/L aqueous suspension, which was further diluted to 1000 mg/L in ultrapure water. GO was produced using the modified Hummers’ method, and detailed analysis of physicochemical properties, including transmission electron microscopy (TEM), scanning electron microscopy (SEM) with elemental point analysis, and Fourier transform infrared spectroscopy, has been provided before by the authors (Strojny et al. 2020). Briefly, the size of the GO flakes was > 5 µm in diameter and up to 1 nm in thickness; the elemental analysis showed that there was > 35% of carbon and > 51% of oxygen. The residues were sodium (7%), sulfur (2%), and chlorine (< 2%). For the experiment on human cell lines, a series of dilutions (10, 50, 100, 125, 250 mg/L) were prepared in ultrapure water after sonification (550 W/m^2^) of 1000 mg/L GO stock in an ultrasonic bath for 30 min (Ultron, Olsztyn, Poland). The final tested concentrations were prepared by mixing the dilution with culture media in a 1:10 ratio (the final concentrations were 1, 5, 10, 12.5, and 25 mg/L). For bacterial cultures, 20 mg/L was prepared and diluted directly in the culture medium in a 1:4 ratio (final concentration 5 mg/L).

Silver nanoparticles (Ag) were obtained from Nanotech (Warsaw, Poland) in the form of a 100 mg/L hydrocolloid. The size distribution with the hydrodynamic diameter was measured by the dynamic light scattering technique using a Zetasizer Nano-ZS90 analyzer (Malvern, Worcestershire, UK). Each measurement was performed in triplicates after 30 s of stabilization at 25 °C. For the experiment on human cell lines, a series of dilutions (10, 20, 30, 40, 50 mg/L) were prepared in ultrapure water after sonication (550 W/m^2^) of the original stock in an ultrasonic bath for 30 min. The final tested concentrations were prepared by mixing the dilution with culture media in a 1:10 ratio (the final concentrations were 1, 2, 3, 4, and 5 mg/L). For part of the experiments, 25 mg/L was additionally prepared (indicated in the further part). For bacterial cultures, the original stock was diluted directly in the culture medium in a 1:4 ratio (final concentration 25 mg/L).

The zeta potential of GO, Ag, and GO:Ag in ultrapure water was determined by the microelectrophoretic method with the Smoluchowski approximation on the same analyzer. Nanomaterials were visualized by TEM (JEM-1220; JEOL, Tokyo, Japan) at 80 keV, with a Morada 11-megapixels camera (Olympus Soft Imaging Solutions, Münster, Germany). Fourier transform infrared spectroscopy (FT-IR) spectra were registered in the middle infrared range of 4000–400 cm^−1^ with the use of a Perkin Elmer System 2000 spectrometer (PerkinElmer Inc., Waltham, MA, USA). Initially, the spectrum of background-containing bands generated by the vibrations and rotations of CO_2_ and H_2_O(g) always present in air was registered. The transition spectrum (ratio of working sample to background signals) of liquid samples was registered with 20 scans and 4 cm^−1^ resolution.

Elemental composition of GO:Ag samples was investigated using the energy-dispersive X-ray spectroscopy (EDS). Aqueous suspensions of GO:Ag complexes were dried on the glass slides under fume hood and the powders were collected with a scalpel. The samples were analysed using a Jeol JSM-6010 PLUS/LV In-Touch Scope scanning electron microscope combined with EDS and 10 kV voltage applied.

#### GO:Ag complex preparation

To obtain the concentrated complex, GO and Ag were mixed in the desired proportions with or without the addition of ultrapure water. Directly before mixing, single nanomaterials (GO and Ag) were exposed to sonication (550 W/m^2^) in an ultrasonic bath for 30 min.

The weight proportion of GO and Ag in the complex was always either 1:1 or 1:5. Briefly, to obtain 10 ml of the concentrated complex GO:Ag 50:10, 5 ml of Ag stock solution (100 mg/L), 0.1 ml of GO stock solution (1000 mg/L), and 4.9 ml of sterile ultrapure water were mixed in a tube in sterile conditions. The tube then was transferred into the ultrasonic bath and exposed to sonication process (550 W/m^2^) for 60 min. To obtain 10 ml of the concentrated complex GO:Ag 50:50, 0.5 ml of GO (1000 mg/L) were added instead of 0.025 ml, and consequently less water (4.5 ml). Before further analyses, the solutions were rested for 30 min in room temperature.

The obtained complex was concentrated either 5 × or 10x (for bacterial cultures and human cell cultures, respectively); therefore it was diluted in the proper media directly before the experiments (as described for GO and Ag in the previous section). The final concentrations in the experimental conditions were GO:Ag 5:25 mg/L (5 mg/L of GO and 25 mg/L of Ag) for bacterial cultures and GO:Ag 5:5 or 1:5 mg/L for human cell cultures.

### Cytotoxicity

#### Cell cultures

Two human-originated cell lines were employed in all the cytotoxicity lines. HFFF2, human fetal foreskin fibroblasts, from the European Collection of Authenticated Cell Cultures, catalogue no 86031405, is a cell line isolated from a 14 to 18 week old male fetus. The cell line has a limited lifespan; passages between 3 and 10 were used for the experiment. A549, lung epithelial cells, from the American Type Culture Collection (ATCC), catalogue no CCL-185™, is a cell line isolated from a 58-year- old carcinoma patient. Passages between 39 and 46 were used for the experiments. Both cell lines are recommended as a model in cytotoxicity screenings.

The HFFF-2 cell line was maintained in Dulbecco’s Modified Eagle Medium (DMEM) with a low glucose concentration (1 g/L), while A549 was maintained in DMEM with a high glucose concentration (4.5 g/L). Both media were supplemented with 10% fetal bovine serum (FBS) and the antibiotics, penicillin (100 U/mL) and streptomycin (100 mg/mL). All media components were purchased from Gibco™; Thermo Fisher Scientific. Cells were cultured in standard conditions (37 °C in a humidified atmosphere with 5% CO_2_). For all experiments, cells were seeded at a density of 1·10^5^ cells/mL.

#### MTT mitochondrial activity assay

Cell viability, expressed as the mitochondrial activity, was assessed by MTT assay, in which yellow water-soluble tetrazolium salt (3-(4,5-dimethylthiazol-2-yl)-2,5-diphenyltetrazolium bromide) is converted into purple formazan crystals in the presence of mitochondrial succinate dehydrogenase. The intensity of the purple color is proportional to the viability of the cells.

Cells were seeded on a 96-well clear-bottom plate (Eppendorf); after overnight incubation, the medium was replaced with medium containing nanomaterials at all the tested concentrations. After 24 h of incubation with the nanomaterials, 15 µL of MTT (Sigma Aldrich) dissolved in PBS (5 mg/mL) were added per well, and after 3 h of incubation at 37 °C, the medium was removed and 100 µL of solubilization solution (90% isopropanol, 10% Triton-X100, 0.01 M HCl) were added per well. Plates were placed on a rotary shaker for 15 min to dissolve the formazan crystals; then, the plates were centrifuged (5 min, 2000 rpm), and the supernatant was transferred to a new microplate. Spectrophotometer readings were performed at 570 nm on a microplate reader (Tecan Group Ltd). Cell viability was expressed as the percentage of control group viability, which was 100%. Calculations were performed with the following formula:

$$Cell\;viability=\frac{{Abs}_{test}}{{Abs}_{control}}\cdot 100\%$$

where ${Abs}_{test}$ is the absorbance of wells exposed to the nanomaterials, and ${Abs}_{control}$ is the mean absorbance of the control wells.

#### Neutral red uptake assay

Cell viability was also assessed using the neutral red assay, in which the dye is taken up by the lysosomes of the viable cells. The amount of the dye incorporated is proportional to the number of viable cells. Before the test, a neutral red (NR) solution was prepared. Then, 0.4 g of NR was dissolved in 100 ml of ultrapure water, and the solution was filtered and heated to 37 °C directly before application. The cells were seeded on a 96-well clear bottom plate (Eppendorf); after overnight incubation, the medium was replaced with medium containing nanomaterials at all tested concentrations. After 24 h of incubation with the nanomaterials, the medium was removed, and cells were washed with PBS. Then, 150 µL of NR solution were added to each well, and the plates were incubated for 3 h at 37 °C. NR solution was then removed, and the cells were dissolved by adding 100 µL of solubilization solution (1% glacial acetic acid solution, 50% ethanol, 49% H_2_O). The plates were placed on a rotary shaker for 15 min to dissolve the cells. Spectrophotometer readings were performed at 540 nm on a microplate reader (Tecan Group Ltd). Cell viability was expressed as the percentage of control group viability, which was 100%. Calculations were performed from with the following formula:

$$Cell\;viability=\frac{{Abs}_{test}}{{Abs}_{control}}\cdot 100\%$$

where ${Abs}_{test}$ is the absorbance of wells exposed to the nanomaterials, and ${Abs}_{control}$ is the mean absorbance of the control wells.

#### LDH leakage assay

Cytotoxicity, expressed as cell membrane damage followed by cytosolic lactate dehydrogenase leakage, was calculated using a Cytotoxicity Detection Kit (LDH) (Roche). LDH released from damaged cells converts a colorless substrate into the red product. The color is proportional to the cytotoxic effect. Cells were seeded on a 96-well clear-bottom plate (Eppendorf); after overnight incubation, the medium was replaced with medium containing nanomaterials at all tested concentrations. An additional control was prepared for the test (maximum LDH) to establish the maximum releasable LDH activity in the cells. After 24 h of incubation with the nanomaterials, the supernatant was transferred to a new plate, and 50 µL of substrate was added to each well;, then, the plates were incubated for 30 min in the dark at room temperature. In the maximum LDH control, cells were dissolved with Triton X-100 for 30 min before the supernatant transfer. Spectrophotometer readings were performed at 490 nm and 690 nm (the reference wavelength) on a microplate reader (Tecan Group Ltd). Cytotoxicity was calculated with the following formula:

$$Cytotoxicity= \frac{test-control}{LDHmax-control}$$

where *test* is the absorbance value of wells exposed to the nanomaterials, *control* is the absorbance value of wells with non-treated cells, and *LDHmax* is the absorbance value of wells with the maximum LDH control.

#### Cell proliferation rate

The proliferation rate of the cells after nanomaterials treatment was determined using a colorimetric immunoassay based on the measurement of 5-bromo-2'-deoxyuridine (BrdU) incorporation in place of thymidine during DNA synthesis (Roche). Cells were seeded on a 96-well clear-bottom plate (Eppendorf); after overnight incubation, the medium was replaced with medium containing nanomaterials at all tested concentrations. After 24 h, 10 µL of BrdU solution were added per well, and then the test was performed in two different modes: cells were incubated with BrdU for either 2 or 24 h, resulting in 26 or 48 h of total incubation time. The labeling medium was then removed, and 200 µL of denaturation/fixation solution were added. Cells were fixed for 30 min at room temperature and then incubated with anti-BrdU antibodies solution for 90 min. The plates were rinsed 3 times with washing buffer, and 100 µL of substrate solution were added per well. After 30 min of incubation at room temperature, spectrophotometer readings were performed at 370 nm and 492 nm (the reference wavelength) on a microplate reader (Tecan Group Ltd). The cell proliferation rate was expressed as the percentage of the control group absorbance, which was 100%. Calculations were performed with the following formula:

$$Cell\;proliferation\;rate=\frac{{Abs}_{test}}{{Abs}_{control}}\cdot 100\%$$

where ${Abs}_{test}$ is the absorbance of wells exposed to the nanomaterials, and ${Abs}_{control}$ is the mean absorbance of the control wells.

#### Reactive oxygen species generation

The level of reactive oxygen species (ROS) formation in cells was determined by a 2′,7′-dichlorofluorescin diacetate (DCF-DA) assay (Sigma Aldrich). DCF-DA is a cell-permeable non-fluorescent probe, which is de-esterified intracellularly and turns into highly fluorescent 2′,7′-dichlorofluorescein upon oxidation. Cells were seeded on a 96-well black plate with a clear bottom (Eppendorf); after overnight incubation, the medium was replaced with medium containing nanomaterials at all tested concentrations. The ROS level was measured after 2 h of cells’ incubation with nanomaterials, when the medium was removed and 100 µL of 10 µM DCF-DA in PBS was added to each well. After 45 min, the solution was removed, and the fluorescence intensity was read at λ = 520 nm after excitation at λ = 480 nm, using a microplate reader (Tecan Group Ltd.). The level of ROS was expressed as the percentage of ROS level in the control group, which was designated as 100%. Calculations were performed with the following formula:

$$ROS\;level=\frac{{MFI}_{test}}{{MFI}_{control}}\cdot 100\%$$

where ${MFI}_{test}$ is the mean fluorescence intensity of wells exposed to the nanomaterials, and ${MFI}_{control}$ is the mean absorbance of control wells.

#### Caspase 3/7 activity

Potential apoptosis induction was measured with CellEvent™ Caspase-3/7 Green Detection Reagent (ThermoFisher Scientific). The reagent is a four-amino acid peptide (DEVD) conjugated to a nucleic acid-binding dye, the sequence of which is a cleavage site for caspase-3/7, and the conjugated dye is non-fluorescent until cleaved from the peptide and bound to DNA. In the presence of caspase 3/7 activity, the nuclei have a bright green fluorescence. The assay was performed in two modes, for a microplate reader and confocal microscopy.

For microplate fluorescence readings, cells were seeded on a 96-well black plate with a clear bottom (Eppendorf); after overnight incubation, the medium was replaced with medium containing nanomaterials in all the tested concentrations. Additional positive controls were prepared (1 µM H_2_O_2_, ketoconazole, miconazole). After 2 h of cells’ incubation with nanomaterials, the treatment medium was removed and the diluted reagent (1 droplet per 1 mL of PBS) was added (100 µL per well). After 30 min, caspase 3/7 activity was measured with a microplate reader (Tecan Group Ltd.); the fluorescence intensity was read at λ = 530 nm after excitation at λ = 502 nm.

For confocal microscopy, imaging cells were seeded on a 6-channel µ-Slide VI 0.4 (Ibidi). After seeding, performed according to the supplier’s recommendation, the slides were incubated over-night, and then the medium was replaced with medium containing nanomaterials at the selected concentrations (0, 1, and 5 mg/L for GO, 5 mg/L for Ag, 5:5 mg/L and 1:5 mg/L for GO:Ag complex). Positive controls were applied as stated above. After 2 h of cells’ incubation with nanomaterials, the treatment medium was removed and the diluted reagent (1 droplet per 1 mL of PBS) was added (30 µL per channel), and the slides were incubated for 30 min. Then, the reagent was removed, and the cells were washed with PBS and fixed for 15 min in 4% paraformaldehyde at room temperature. Next, cells were counterstained with 300 µM DAPI and phalloidin conjugated with Alexa Fluor 633 (both Molecular Probes), dissolved in PBS, for 30 min, for nuclei and cytoskeleton imaging. After washing with PBS and deionized water, the channels were filled with Fluoromount Aqueous Mounting Medium (Sigma Aldrich), and cells were visualized with a confocal microscope FV-100 (Olympus, Tokyo, Japan).

#### Cell morphology analysis

The general morphology of the cells was recorded either for living cells using the contrast phase or for fixed cells after May–Grünwald and Giemsa staining. Two approaches were applied: either using the standard method, where nanomaterials were added after cells’ adhesion to the culture plate surface, or seeding cells on plates pre-coated with nanomaterials.

Pre-coated plates were prepared by distributing 1 mL of nanomaterials in the selected concentrations (ultrapure water as a vehicle control; 1, 5, and 25 mg/L of Ag or GO; 50:50 and 10:50 mg/L of GO:Ag) evenly at the bottom of 12-well plates (Falcon), and the plates were dried overnight in sterile conditions in a laminar-flow chamber. Cells were then seeded and incubated for 24 h. In the second method, cells were seeded first; then, after overnight incubation, the medium was replaced with medium with nanomaterials at the same concentrations, and cells were incubated for 24 h or 7 days.

For staining, the medium was removed, and the cells were washed gently with PBS. Then, 1 mL of May–Grünwald stain (Sigma Aldrich) was added; after 3 min, 1 mL of PBS was added to the wells. After 3 additional minutes, the staining solution was replaced with diluted Giemsa solution (1:20 in deionized water). After 20 min, the cells were washed thoroughly with deionized water and dried for further examination. Imaging was performed using a CKX41 inverted microscope (Olympus).

### Antibacterial activity

The antibacterial properties of GO, Ag, and the GO:Ag complex were analyzed with the use of two bacterial strains. *Staphylococcus aureus* (no. 25923) and *Pseudomonas aeruginosa* (no. 27853) were obtained from the ATCC. The strains were stored in suspensions in 20% (v/v) glycerol at − 20 °C. Prior to their use in experiments, the strains were defrosted, and the glycerol was removed by washing the bacterial cells with distilled water. The bacteria were grown on nutrient agar (Biomaxima, Lublin, Poland). After 24 h of incubation on solid medium, the bacteria were inoculated into Mueller–Hinton (MH) broth at 37 °C. The bacterial concentration was determined by measuring the optical density at 600 nm. Briefly, bacterial suspensions were prepared from overnight cultures and adjusted to 10^6^ CFU/mL. Aqueous suspensions of GO, Ag, and the GO:Ag complex in concentrations of 20 mg/L GO, 100 mg/L Ag, and GO:Ag 20:100 mg/L were prepared and added to bacterial suspensions in a 1:4 ratio (final concentrations: 5 mg/L GO, 25 mg/L Ag, GO:Ag 5:25 mg/L). The suspensions were incubated for 24 h at 37°C. Control samples of bacteria were treated with ultrapure water. After incubation, serial tenfold dilutions (up to 10^−6^) were prepared. One hundred microliters of each dilution were transferred to Petri dishes with nutrient medium, and after 24 h of incubation at 37 °C, the number of colonies formed was calculated. All incubations were conducted in triplicate. Based on the results of the plate counts, the number of bacteria (CFU, colony forming units) was determined in each of the samples and controls.

#### Minimum inhibitory concentration and minimum bactericidal concentration

To define minimal inhibitory concentration (MIC) of nanomaterials used, 24-well plate was filled with the Mueller Hinton medium (BioMaxima, Lublin, Poland). Thereafter, nanomaterials suspensions were added to the medium to a final concentration of Ag: 25; 12.5; 6.25; 3.125; 1.56; 0.78 mg/L; GO: 5; 2.5; 1.25; 0.625; 0.3125 mg/L, and their combination (Ag 25 mg/L + GO 5 mg/L; Ag 12.5 mg/L + GO 2.5 mg/L; Ag 6.25 mg/L + GO 1.25 mg/L; Ag 3.125 mg/L + GO 0.625 mg/mL; Ag 1.56 mg/mL + GO 0.625 mg/L; Ag 0.78 mg/L + GO 0.3125 mg/L). Then bacterial suspensions (0.5 on a McFarland scale) were added into the wells in a volume of 10 µL. The experiment was conducted in the presence of positive control (medium with bacterial suspension without the addition of nanoparticles) and negative control (medium without bacterial suspension). Plates were incubated for 24 h at 37 °C. Results were determined as the lowest concentration of the nanomaterials that visibly inhibited growth after the incubation.

In order to define minimum bactericidal concentration (MBC) of nanomaterials used, 10 µL of samples from each well were spread onto Mueller Hinton agar (BioMaxima, Lublin, Poland) and Petri dishes were incubated as above. Results were defined as the lowest concentration that inhibits bacterial growth on the agar plates surface.

#### Presto blue viability assay

To determine dose-dependent influence of Ag, GO and GO:Ag on bacteria viability, 90 µl of bacteria suspensions (with or without nanomaterials) from MIC assay were transferred to 96-well plates. 10 µl of Presto Blue (Thermo Scientific) were added to each well and after 20 min incubation at 37°C, absorbance was read at 570 nm, with 600 nm reference wave. Cell viability was calculated using the following formula:

$$Cell\;viability=\frac{{Abs}_{test}}{{Abs}_{control}}\cdot 100\%$$

where ${Abs}_{test}$ is the absorbance of wells exposed to the nanomaterials, and ${Abs}_{control}$ is the mean absorbance of the control wells (bacterial suspension not treated with nanomaterials). ${Abs}_{test}$ and ${Abs}_{control}$ where corrected by their respective blank wells (suspensions without bacteria).

### Statistical analysis

Where appropriate, data were analyzed using one-factorial analysis of variance followed by Tukey’s multiple comparisons test. Differences with *p* < 0.05 were considered significant. Analysis was performed using GraphPad Prism 9.1.2 (GraphPad Software, San Diego, California USA).

## Results

### Nanomaterials

**Fig. 1:**
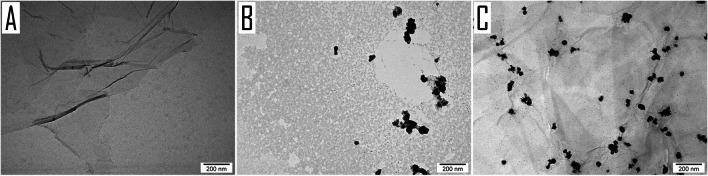
Visualization of GO 5 mg/L (**A**), Ag 25 mg/L (**B**), and GO:Ag 5:25 mg/L (**C**) by TEM. Scale bar 200 nm

The representative morphology of GO, Ag, and the GO:Ag complex is presented in Fig. 1. Ag particles were spherical to oval in shape, ranging in size between 5 and 35 nm. In the TEM images, it is noticeable that smaller nanoparticles were evenly distributed on the GO flakes, while larger ones formed agglomerates, which were also fairly distributed.

Zeta potential analysis showed that all tested nanomaterials have a highly negative zeta potential, ranging from approximately – 35 to − 50 mV (Table 1), which indicates good stability of the GO, Ag, and GO:Ag hydrocolloids that were prepared in ultrapure water. The measurements confirmed our visual observation of the prepared stocks during storage – none of the prepared nanomaterials sedimented significantly.

**Table 1:** Mean zeta potential of GO, Ag, and GO:Ag complexes

Group	GO (10 mg/L)	GO (50 mg/L)	Ag (50 mg/L)	GO:Ag (50:50 mg/L)	GO:Ag (10:50 mg/L)
Zeta potential	− 44 ± 0.5 mV	− 35 ± 2.2 mV	− 51 ± 1.4 mV	− 50 ± 0.6 mV	− 35 ± 1.6 mV
Mean hydrodynamic diameter	896 ± 55.7 nm	1345 ± 31.8 nm	109 ± 1.9 nm	127 ± 1.8 nm	256 ± 12.9 nm
Polydispersity index	0.60 ± 0.011	0.47 ± 0.053	0.28 ± 0.025	0.52 ± 0.028	0.52 ± 0.034

**Fig. 2:**
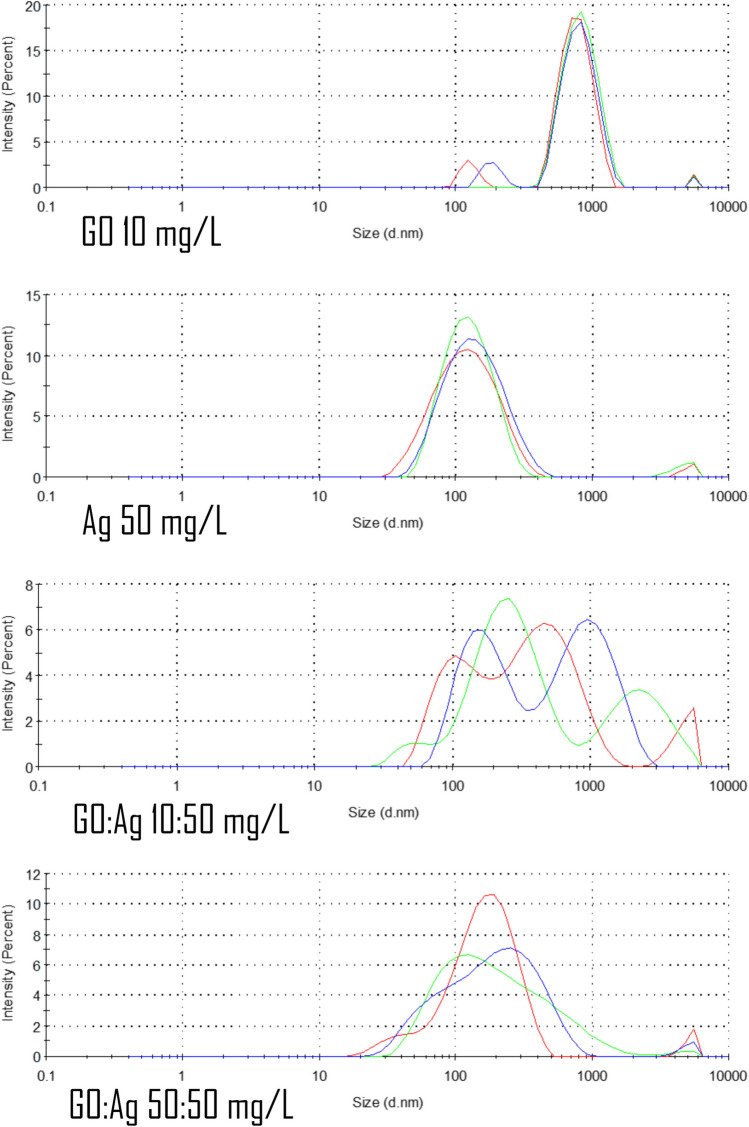
Size distribution of GO, Ag, and GO:Ag complexes determined by DLS method. Different colors represent measurements in triplicate

DLS analysis of GO, Ag, and Ag:GO confirmed the formation of the agglomerates of different sizes (Fig. 2). We present the size distribution, mean hydrodynamic diameter, and polydispersity index (Table 1). Although the DLS method is not suitable for size determination of non-spherical nanomaterials (such as GO), nevertheless, it provides an insight into the size distribution and agglomerates’ formation in the case of Ag. We added the GO for comparison. Interestingly, mixing the GO and Ag in higher, equal concentrations (50:50 mg/L) seems to have a positive impact on the final nanomaterial’s stability, where we observed lowered zeta potential and hydrodynamic diameter; additionally, the size distribution gave more stable results during DLS analysis.

**Fig. 3:**
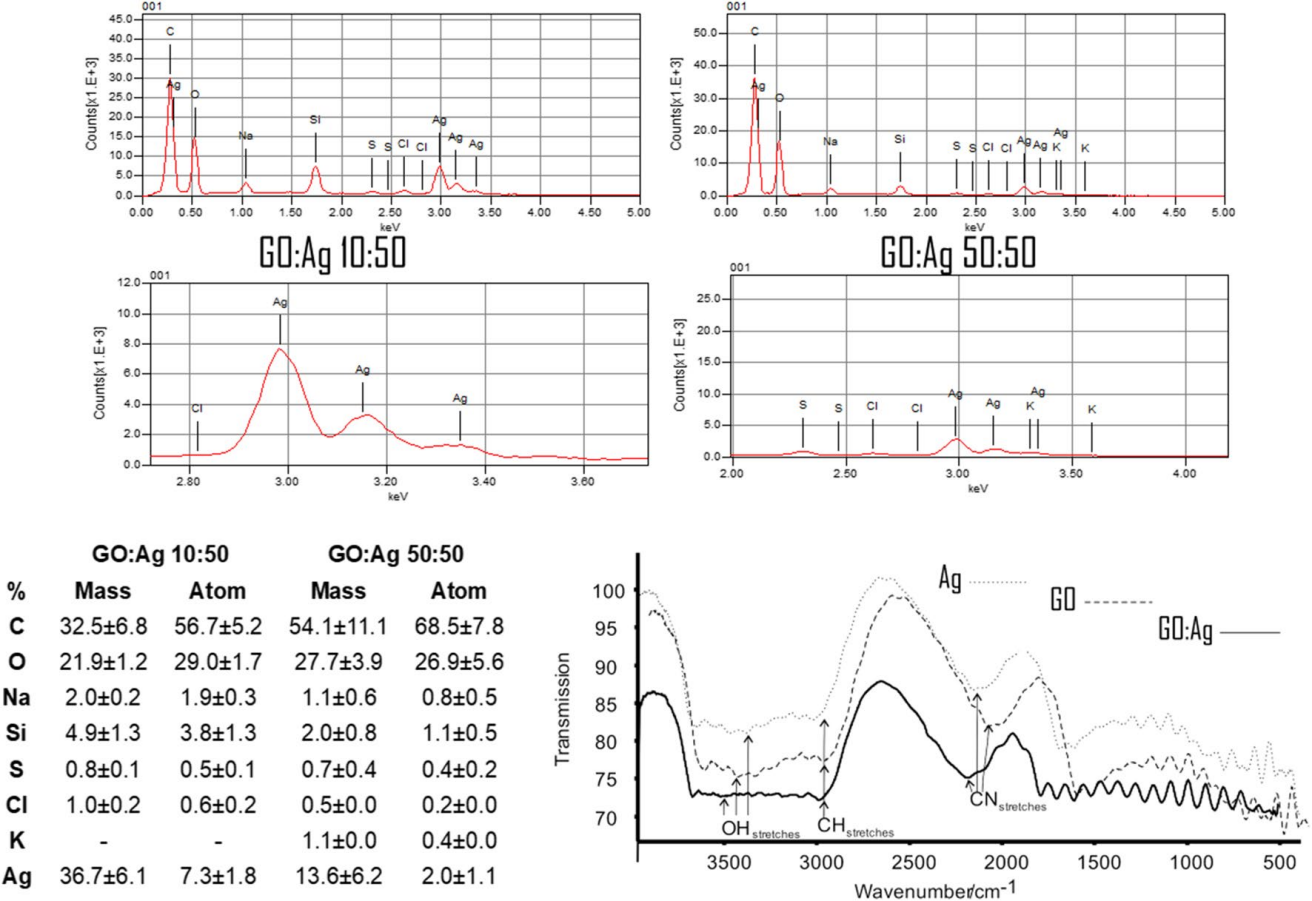
Spectral of elemental analysis of the GO:Ag complexes performed by energy-dispersive X-ray spectroscopy (EDS) and Fourier transform infrared spectroscopy (FT-IR) spectra of Ag, GO and GO:Ag complex. Calculations for EDS were performed from four replicates

An estimated mass concentration of elemental analysis performed by EDS for GO:Ag complexes was: 32.5% and 54.1% of carbon; 21.9% and 27.7% of oxygen; 36.7% and 13.6% of silver, in 10:50 and 50:50 complex, respectively. In both complexes sodium, chlorine, and potassium were detected below 2% of total mass. Detected silicon was an impurity resulting from the sample preparation. All results, both as mass and atom percentage are presented on Fig. 3. FT-IR spectra analysis (Fig. 3) revealed that the GO:Ag complex has some of the band characteristics of the single components, GO and Ag, but also new unique characteristics. We observed characteristic bands for Ag in the regions 1686 cm^−1^ and 1606 cm^−1^, and for GO in the regions of 1649 cm^−1^ and 1700 cm^−1^. In the GO:Ag complex, the 1649 cm^−1^ band from GO was still present, but the one in the 1700 cm^−1^ region was missing; similarly, the band in the 1686 cm^−1^ region from Ag was missing. A new band in the spectral region of 1610 cm^−1^, which was not present in Ag or GO, appeared. In the case of the bare Ag nanoparticles, a wide band corresponding to -OH groups was present, indicating hydrogen bonds’ occurrence, probably with the water molecules. Meanwhile, in the GO:Ag complex, the band was narrow, indicating limited interactions with water molecules, which may have an impact on the improved and enhanced properties of Ag in the complex with GO, which neutralizes Ag agglomeration, helps with even Ag distribution, and provides better contact with the cells.

### Cytotoxicity

**Fig. 4:**
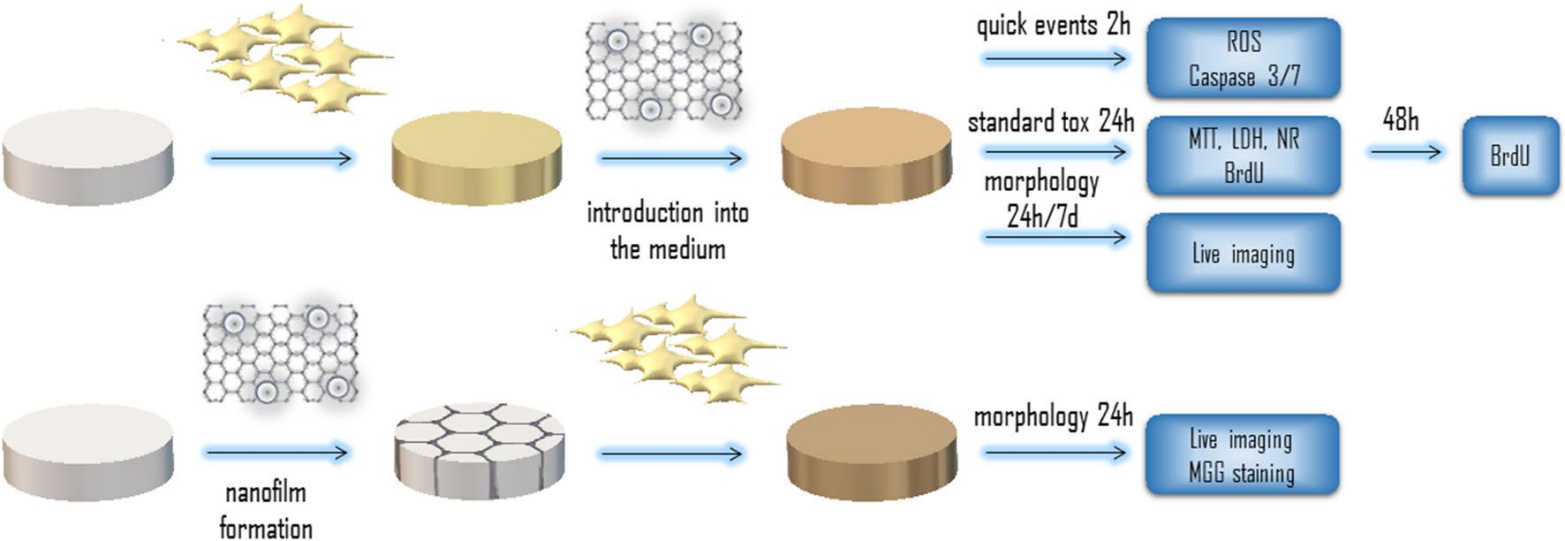
Schematic representation of the cytotoxicity panel. A series of cytotoxicity evaluations were performed using a standard method, where nanomaterials were added after cells’ adhesion to the culture plate surface. A quick cellular response was evaluated by measuring ROS level and Caspase 3/7 activity combined with confocal microscopy imaging. Cell viability (measured by MTT, LDH and NR assays) was evaluated after a standard 24 h incubation time, while proliferation was additionally measured after 48 h. Cell growth characteristics and their morphology was evaluated additionally after 7 days of incubation with the nanomaterials. A second approach was applied for morphology imaging, where cells were seeded on plates pre-coated with nanomaterials

**Fig. 5:**
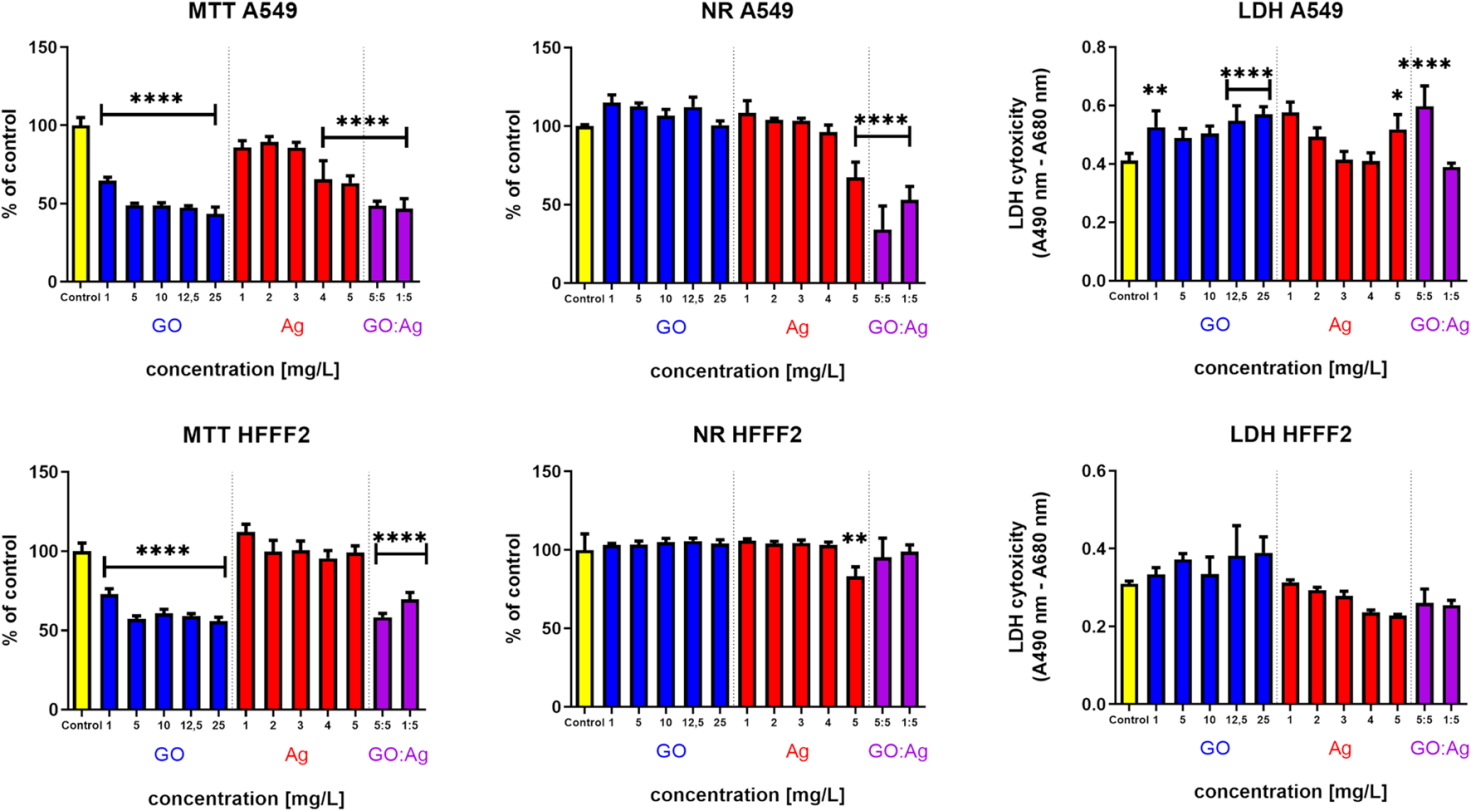
A549 and HFFF2 cells viability 24 h after incubation with nanomaterials, measured by three different assays. Statistically significant difference between a group and the control is marked as * *p* < 0.05, ** *p* < 0.0021, *** *p* < 0.0002, p**** < 0.0001. MTT and NR results are presented as % of control (mean with standard variation). Blue bars, GO; red bars, Ag; purple, GO:Ag. Notes: MTT is based on mitochondrial succinate dehydrogenase activity; NR is based on lysosomal uptake of neutral red. LDH is based on leakage of cytosol lactic dehydrogenase

We performed three viability tests, relying on three different mechanisms, namely MTT, based on the mitochondrial succinate dehydrogenase activity; NR, based on the neutral red uptake by the lysosomes of the living cells; and LDH, based on the activity of the cytosol lactate dehydrogenase leakage through the damaged cell membrane (schematic representation of the cytotoxicity panel is presented in the Fig. 4). The results differed significantly between the tests; nevertheless, they suggested lower vulnerability of HFFF2 to damage in the presence of nanomaterials (Fig. 5). While the MTT results suggest high toxicity of GO (both alone and in the GO:Ag complex, the decrease reached 50% in relation to the control group), the NR results do not confirm this for bare components. Similarly, for LDH leakage, even though there were statistically significant differences in the results, these were not confirmed by those of MTT or NR. These findings, together with the following results and our previous observations, suggest rather false negative results from the MTT test, where GO itself probably inhibits the turnover of formazan by succinate dehydrogenase. We previously observed that GO can have significance in inhibiting enzymatic reactions inhibition (Strojny et al. 2020). Neutral red, which is a supravital dye incorporated by the lysosomes of viable cells and thus is not based on biochemical turnover of the reagents, seems to be less prone to such interactions. Nevertheless, the GO:Ag complex in the concentration 5:5 mg/L had a significant cytotoxic effect on the A549 cells, but not on HFFF2 cells.

#### Cell proliferation rate

**Fig. 6:**
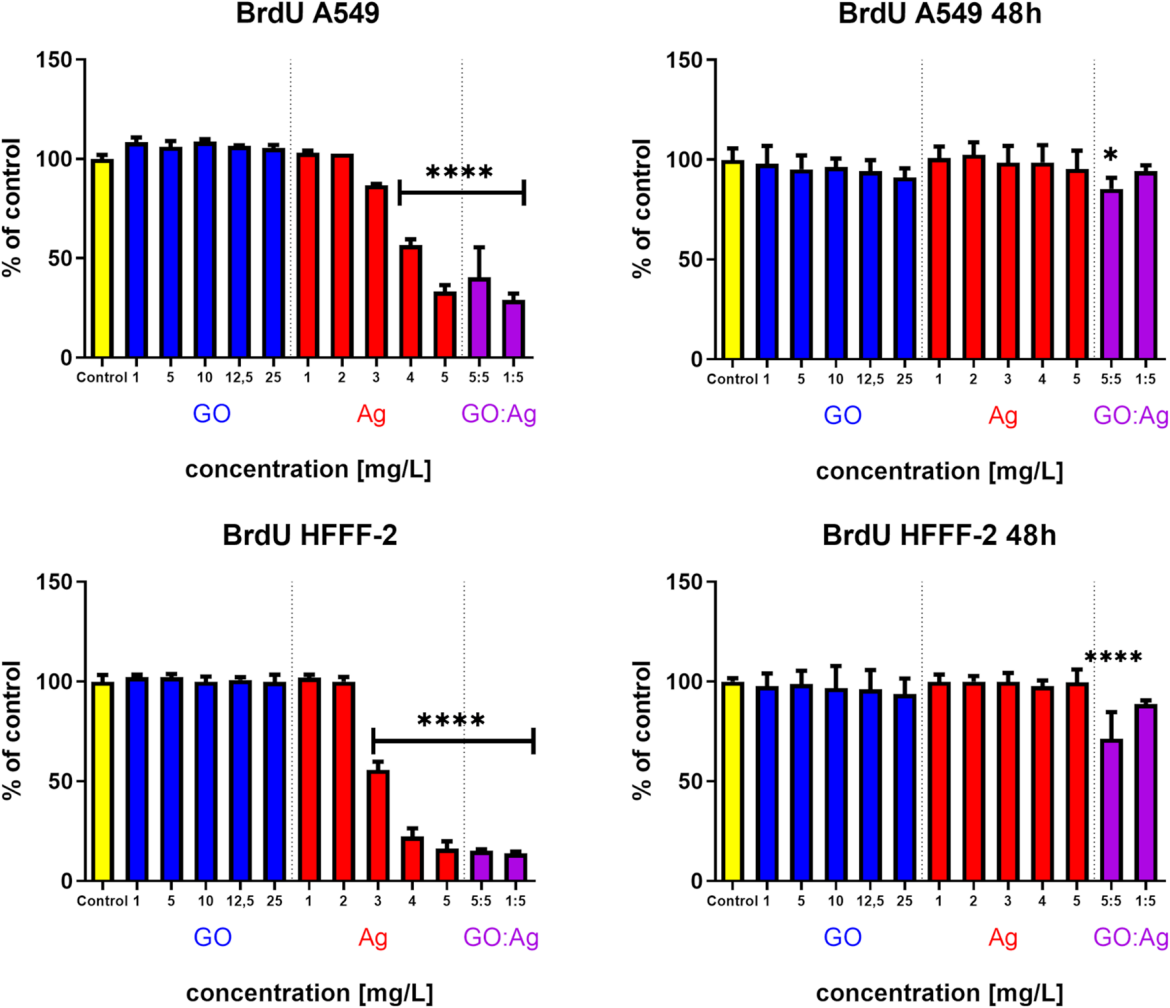
A549 and HFFF2 cell proliferation rate after 24 h and 48 h of incubation with nanomaterials, measured by colorimetric BrdU assay. Statistically significant difference between a group and the control is marked as * *p* < 0.05, **** *p* < 0.0001. Results are presented as % of control (mean with standard variation). Blue bars, GO; red bars, Ag; purple, GO:Ag

GO had no effect on proliferation in either cell line, while Ag inhibited the proliferation rate in both cell lines in the a dose-dependent manner (Fig. 6). The proliferation rate of A549 cells in relation to the control group dropped to 86%, 56%, and 33% in the Ag groups with 3 mg/L, 4 mg/L, and 5 mg/L, respectively. In HFFF2 cells, the proliferation rate dropped to 55%, 22%, and 16% in the Ag groups with 3 mg/L, 4 mg/L, and 5 mg/L, respectively. GO:Ag inhibited the proliferation very significantly in both cell lines as well: in the GO:Ag 5:5 mg/L group reached 40% of the control in A549 cells and 15% in HFFF2 cells, while the GO:Ag 1:5 mg/L group, reached 15% in A549 and 30% in HFFF2 cells. However, the effect was visible only in the case of 24-h treatment with the nanomaterials (2 h of incubation with BrdU compound); after 48-h incubation with nanomaterials (and 24 h of incubation with BrdU compound), the effect caused by Ag was gone, while GO:Ag at a concentration of 5:5 mg/L still inhibited proliferation significantly, but the effect was not as strong as after 24 h (85% of control in A549 cells and 71% of control in HFFF2 cells).

#### Reactive oxygen species generation

**Fig. 7:**
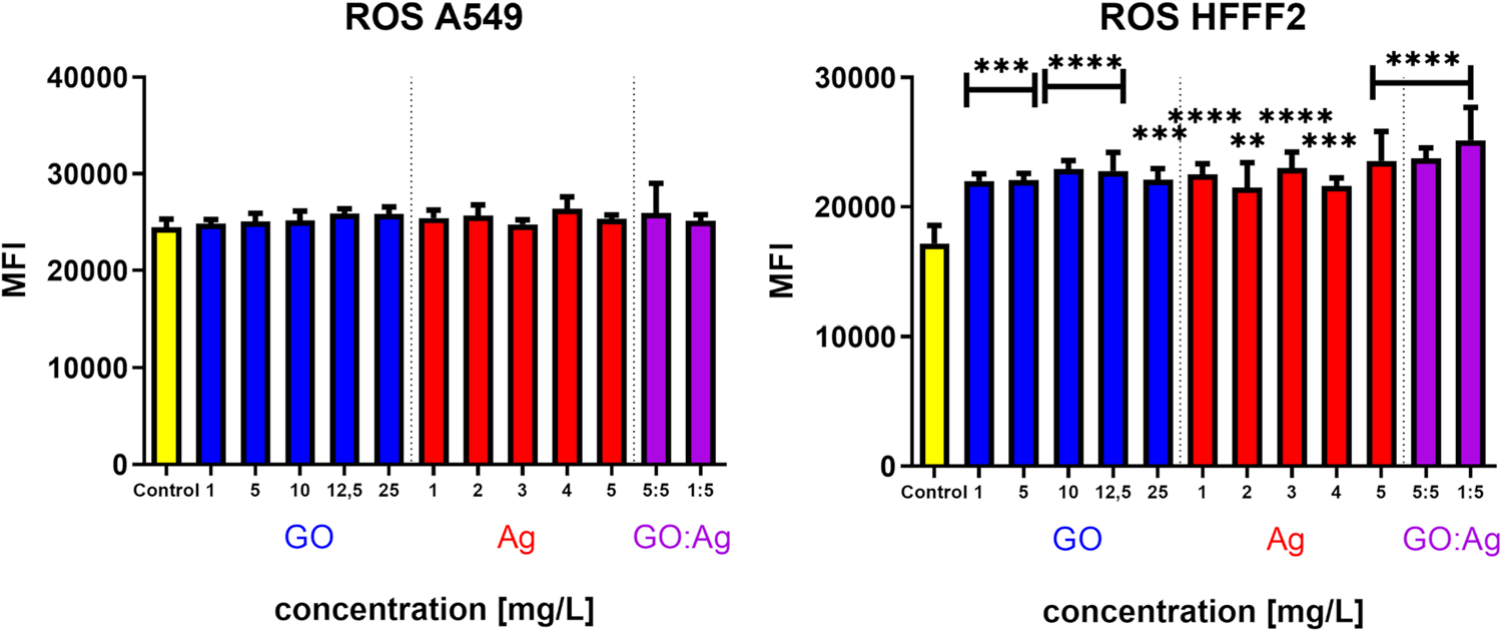
Reactive oxygen species (ROS) level in A549 and HFFF2 cells after 2 h of incubation with nanomaterials, measured by colorimetric DCF-DA fluorometric assay. Statistically significant difference between a group and the control is marked as ** *p* < 0.0021, *** *p* < 0.0002, p**** < 0.0001. Results are presented as mean fluorescence intensity (MFI) with standard deviation. Blue bars, GO; red bars, Ag; purple, GO:Ag

None of the tested nanomaterials had an effect on A549 cells, while in the case of HFFF2 cells, all of the nanomaterials at all the concentrations induced ROS generation (Fig. 7). The effect was rather equal and nondose dependent.

#### Caspase 3/7 activity

**Fig. 8:**
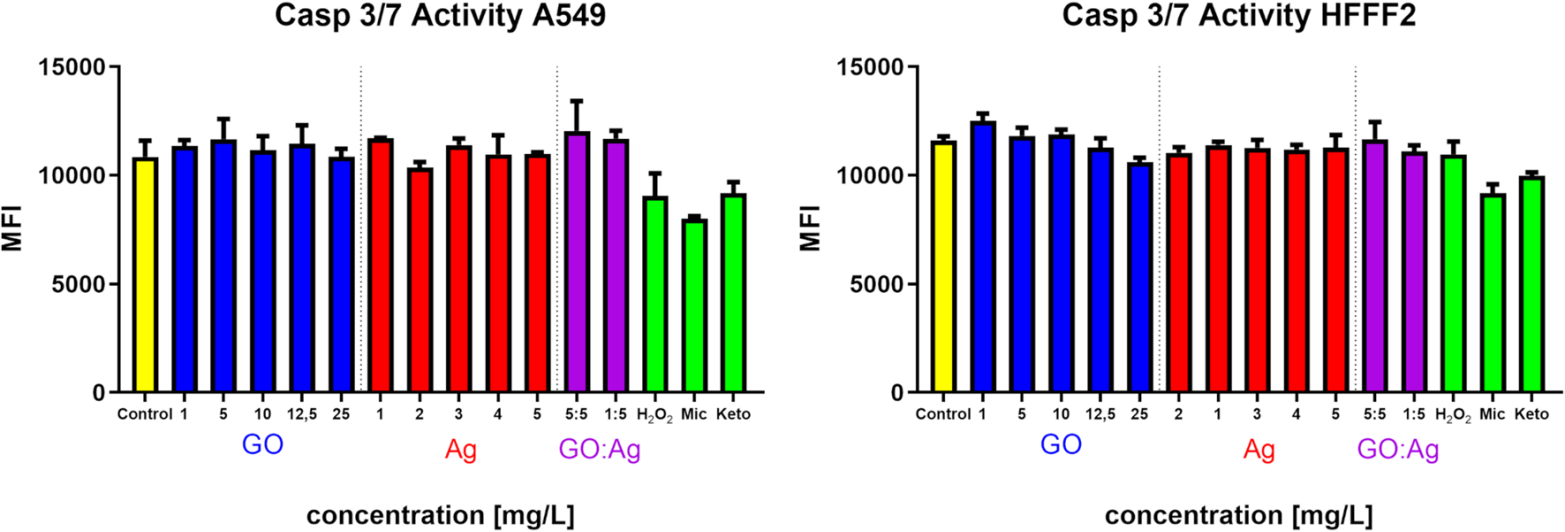
Caspase 3/7 activity in A549 and HFFF2 cells after 2 h of treatment with nanomaterials, measured by fluorometric CellEvent assay. There were no statistically significant differences. Results are presented as mean fluorescence intensity (MFI) with standard deviation. Blue bars, GO; red bars, Ag; purple, GO:Ag; green, additional controls

**Fig. 9:**
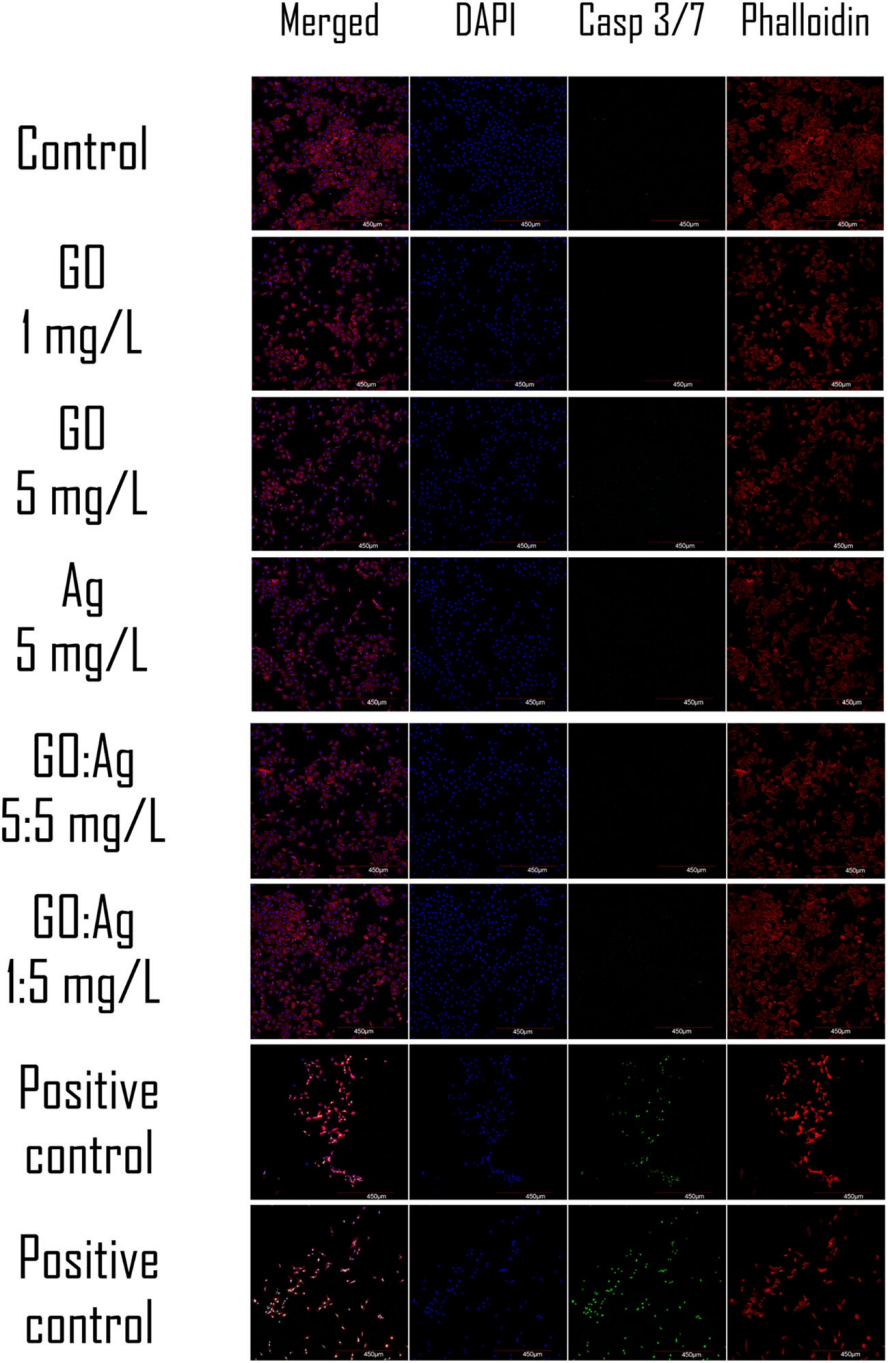
Confocal imaging of A549 cells combined with caspase 3/7 activity detection after treatment with nanomaterials for 2 h. Additional positive control: miconazole. Scale bar 450 µm. Notes: Nuclei were stained with DAPI (blue); caspase 3/7 activity was visualized using a CellEvent probe (green), which stains nuclei in the presence of active caspases; actin F was stained with phalloidin conjugated with Alexa Fluor 633 (red)

**Fig. 10:**
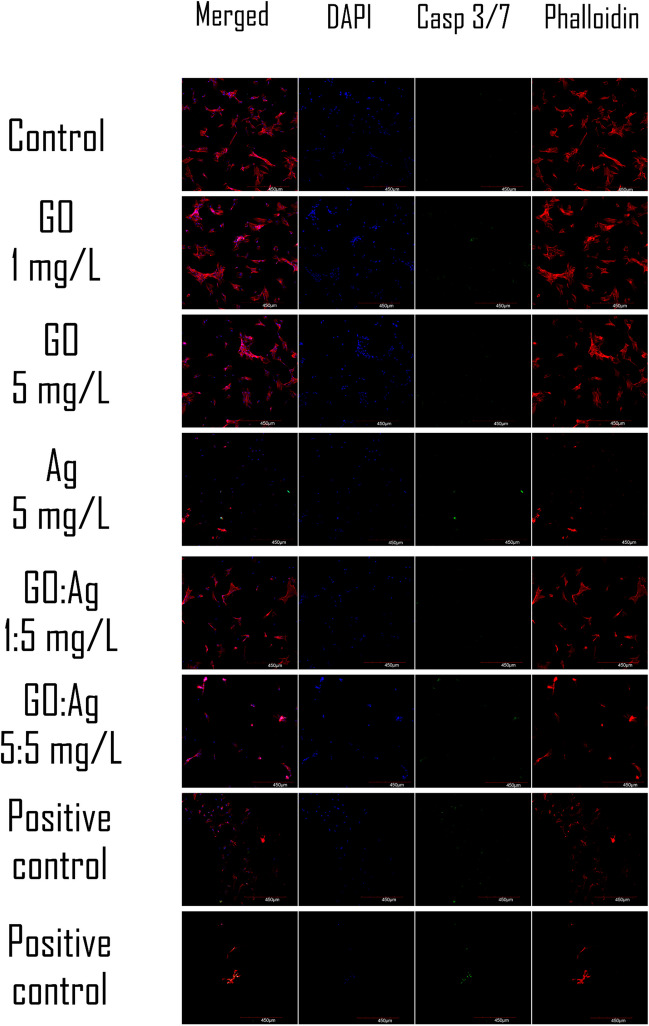
Confocal imaging of HFFF2 cells combined with caspase 3/7 activity detection after treatment with nanomaterials for 2 h. Additional positive control: ketoconazole. Scale bar 450 µm. Notes: Nuclei were stained with DAPI (blue), caspase- 3/7 activity was visualized using a CellEvent probe (green) which stains nuclei in the presence of active caspases; actin F was stained with phalloidin conjugated with Alexa Fluor 633 (red)

**Fig. 11:**
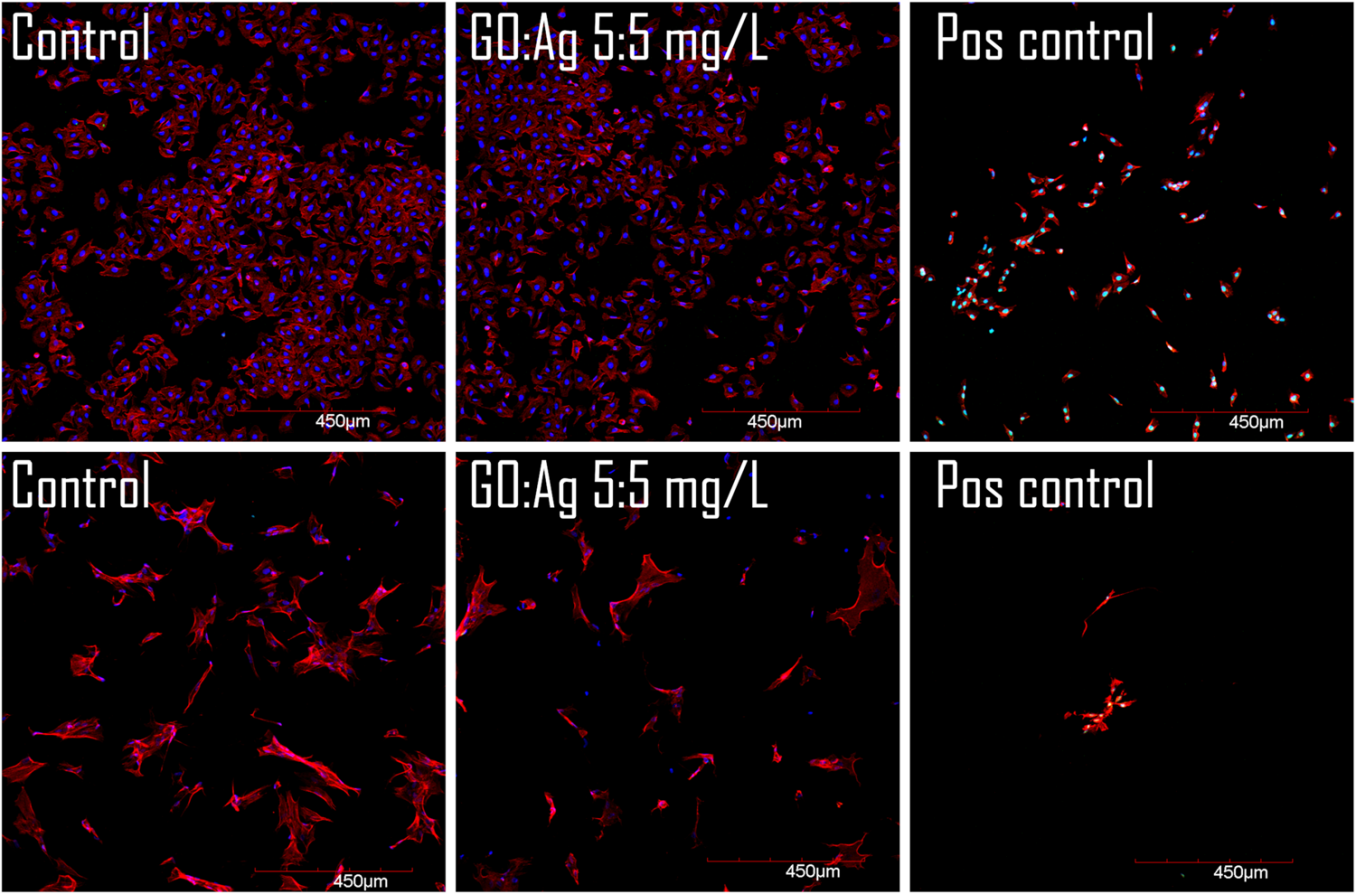
Selected magnified pictures from confocal imaging of A549 and HFFF2 cells combined with caspase- 3/7 activity detection after treatment with nanomaterials for 2 h. Additional positive controls: miconazole (A549) and ketoconazole (HFFF2). Scale bar 450 µm. Notes: Nuclei were stained with DAPI (blue); caspase 3/7 activity was visualized using a CellEvent probe (green, bright nuclei) which stains nuclei in the presence of active caspases; actin F was stained with phalloidin conjugated with Alexa Fluor 633 (red)

Caspase- 3/7 activity, which is an early indicator of apoptosis, was not detected in the GO, Ag, or GO:Ag groups in fluorometric assay (Fig. 8) or in confocal microscopy imaging (Figs. 9 and 10) for both cell lines, except Ag 5 mg/L in the HFFF2 cell line, where a weak signal was recorded during confocal imaging. In this case, the density of the cell culture was lowered in the 5 mg/L Ag group, and likewise in the 1:5 mg/L GO:Ag group. The cytoskeleton, visualized by the phalloidin conjugate, was condensed, and the cells were shrunken in these groups. Interestingly, the effect was not visible at the higher concentration of GO in the complex (GO:Ag 5:5 mg/L). In A549 cells, in all groups, the cell density was at a similar level, and the cytoskeleton was not disrupted. Bright green fluorescence at the nuclei sites, indicating on the caspase- 3/7 activity, was recorded in positive controls, treated with H_2_O_2_, miconazole, or ketoconazole (Fig. 11).

#### Cell morphology analysis

Cell morphology was recorded either 24 h after standard treatment with the nanomaterials, where the nanomaterials were added to the culturing media, or after seeding the cells on the nanomaterials-coated cell culture multi-well plates. In the second approach, we additionally increased the concentration of the nanomaterials up to 25 mg/L to determine whether cells were able to grow on the GO- and Ag- coated surfaces and retain their basic morphology.

**Fig. 12:**
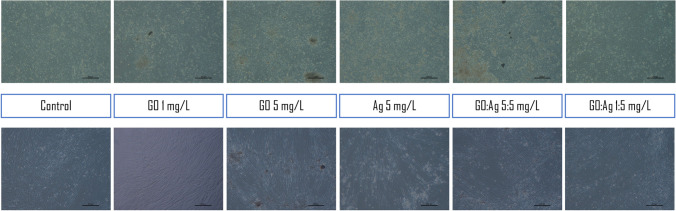
Live imaging of A549 (upper row) and HFFF2 (lower row) cells after 7 days of treatment with nanomaterials, which were introduced into the culture medium after cell adhesion

In the traditional approach, where we added the nanomaterials to the medium, we did not observe visible effects after 24 h in any of the groups. Interestingly, when we prolonged the incubation period for an additional 7 days (Fig. 12), we observed that growth and morphology of both cell lines were unaffected in the groups containing GO.

Even though the density and viability of the cells was decreased in the presence of bare Ag, the growth was unaffected in the presence of the GO:Ag complex. The effect was especially visible in the A549 cell line, where in the presence of the GO:Ag complex at a 5:5 mg/L concentration, the cell density was significantly increased in comparison to the GO:Ag complex at a 1:5 mg/L concentration, suggesting a positive effect of GO on the cell adhesion. The fibroblasts of the HFFF2 cell line were visibly less prone to the potential toxic effects than those of the A549 cells. Both in the control and in all of the GO- containing groups, cells’ density was high, and they created characteristic structures composed of over-lapping cells.

**Fig. 13:**
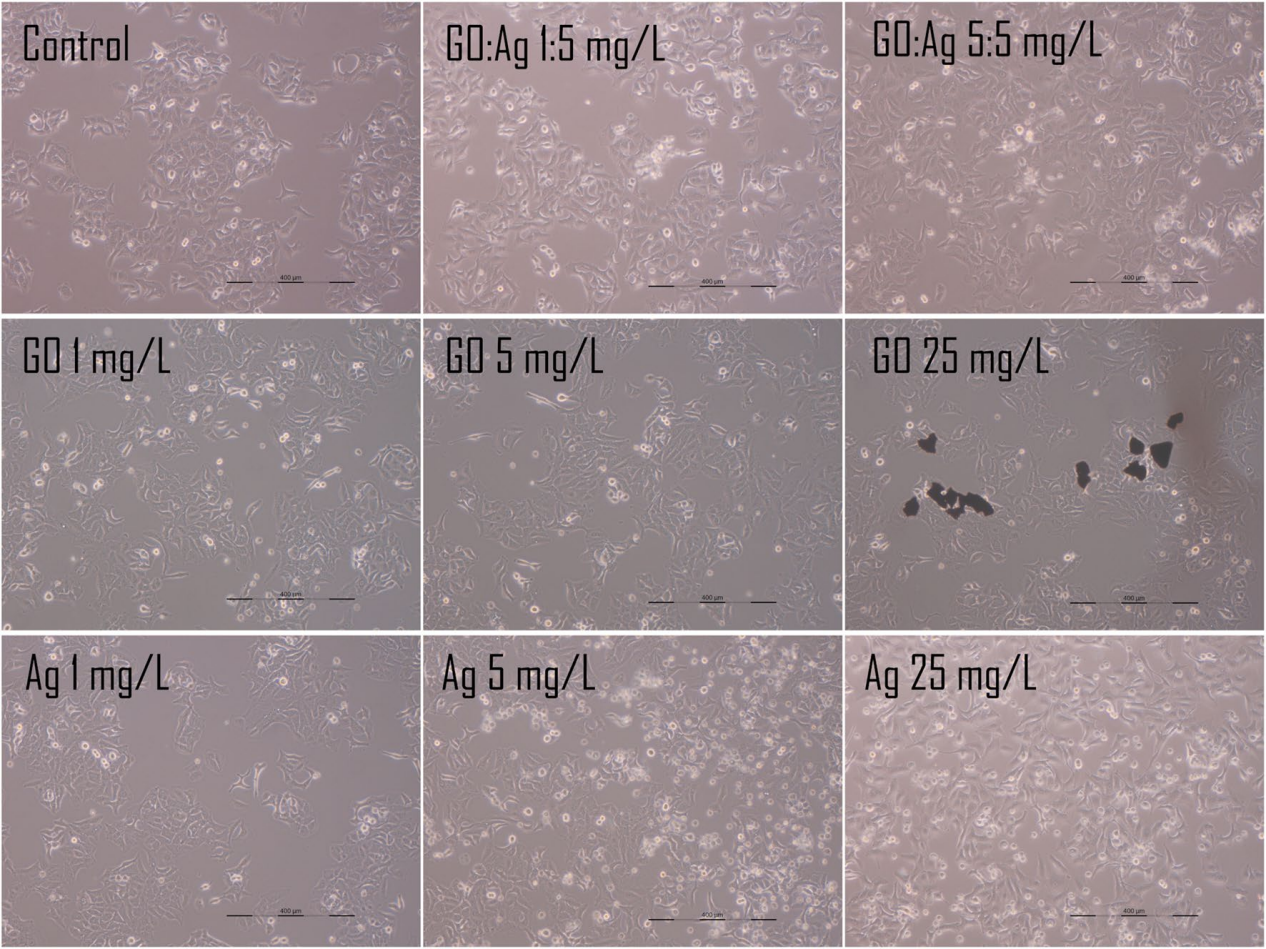
Live imaging of A549 cells after seeding on tissue culture plates coated with nanomaterials. Cells were grown for 24 h. Agglomerates of thicker GO flakes are visible. Scale bar 200 µm

**Fig. 14:**
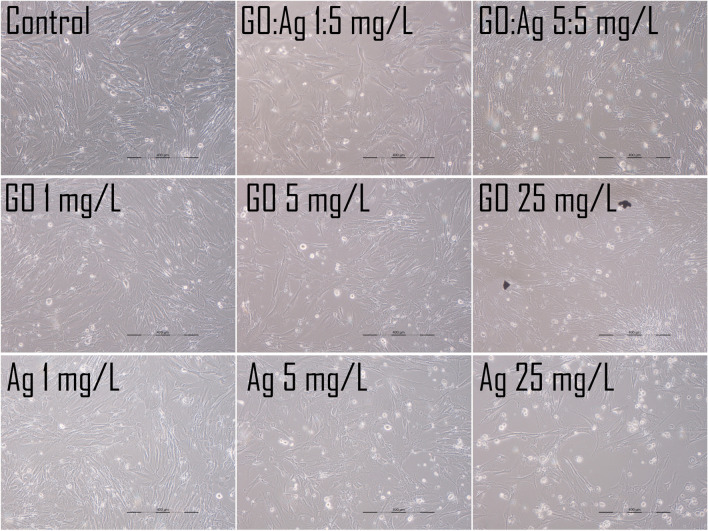
Live imaging of HFFF2 cells after seeding on tissue culture plates coated with nanomaterials. Cells were grown for 24 h. Agglomerates of thicker GO flakes are visible. Scale bar 200 µm

**Fig. 15:**
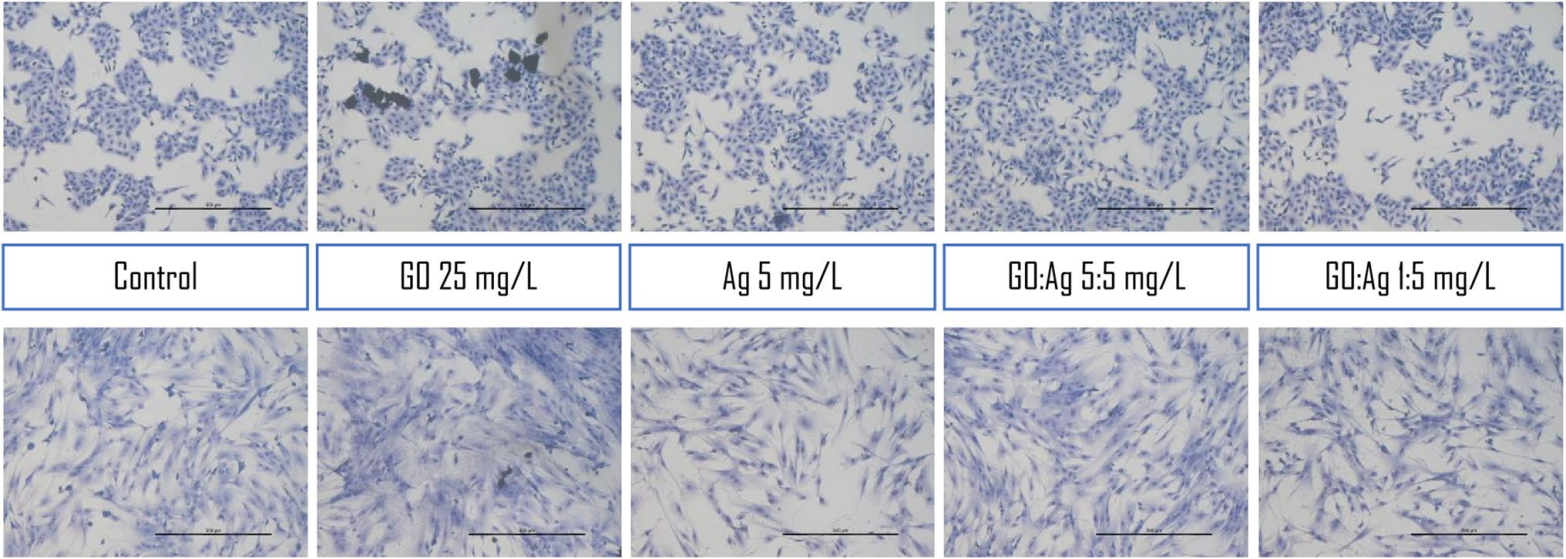
May–Grünwald and Giemsa staining of A549 (upper row) and HFFF2 (lower row) cells after 24 days after seeding on tissue culture plates coated with nanomaterials (selected images). Agglomerates of thicker GO flakes are visible in the A549 cell line; stained large and thin flakes are visible in the HFFF2 cell line. Scale bar 200 µm

In the case of nanomaterials-coated plates, we observed an increased density of A549 cells on Ag-coated plates (more cells were visible, and some were detached; however the surface was fully overgrown, as in the control); cells grew even on the high concentration (25 mg/L) of Ag (Fig. 13), which was completely toxic to the cells when added to the medium, so no tests could be performed at such high concentrations. Effective growth was visible in the A549 cells, while for the HFFF2 cells, the density was visibly lower than that in the control group (Figs. 14 and 15). In the case of the GO coating, we observed increased attachment of the cells to the GO:Ag complex. It was visible that the number of the detached cells in GO:Ag 5:5 mg/L was lower than in the comparable wells containing only Ag.

### Antibacterial activity

**Fig. 16:**
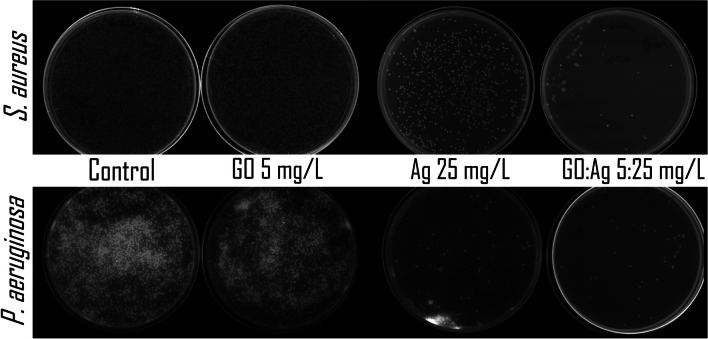
Representative pictures of bacterial culture plates after introduction of GO, Ag, and GO:Ag into the nutrient agar. Incubation time was 24 h. Upper row: *S. aureus*; lower row: *P. aeruginosa*. Notes: Pictures were taken under UV light; fluorescence of compounds secreted by *P. aeruginosa* colonies is visible

GO itself did not influence bacterial growth (Fig. 16, Table 2). A significantly lower number of colony-forming units (CFUs) was observed in the Ag and GO:Ag groups for both bacteria (Table 2). The lowest CFU was obtained in the GO:Ag group (2.0 ± 0.46∙10^3^ in *S. aureus*; 2.4 ± 0.55∙10^4^ in *P. aeruginosa*), even though we did not observe any effect in GO, suggesting a novel effect of the composite in comparison to pure Ag (5.1 ± 0.53∙10^3^ in *S. aureus*, 7.8 ± 1.14∙10^4^ in *P. aeruginosa*). A greater reduction in the bacterial number after Ag and GO:Ag treatment was observed for *S. aureus*. The results were confirmed by MIC of GO:Ag, which was lower for *S. aureus* (0.3125:1.56 mg/L) in comparison to *P. aeruginosa* (0.625:3.125 mg/L). MIC of pure Ag was 3.125 mg/L for both species. MBC of GO:Ag was again lower for *S. aureus* (2.5:12.5 mg/L in comparison to 5:25 mg/L for *P. aeruginosa*), while pure Ag was 12.5 mg/L for *S. aureus* and 25 mg/L for *P. aeruginosa*. Pure GO did not affect bacteria at any given concentration. MIC and MBC results are presented in the Table 3.

**Table 2:** Colony-forming units of *S. aureus* and *P. aeruginosa* after adding GO, Ag, and GO:Ag to the nutrient agar

	*S. aureus*	*P. aeruginosa*
Control	3.0±1.03·10^8^	4.8±0.75·10^8^
GO (5 mg/L)	2.9±0.75·10^8^	4.3±0.36·10^8^
Ag (25 mg/L)	5.1±0.53·10^3^*#	7.8±1.14·10^4^*#
GO:Ag (5:25 mg/L)	2.0±0.46·10^3^*#	2.4±0.55·10^4^*#

^*^ results significantly different from the control group, # results significantly different from the GO group

**Table 3:** Minimal inhibitory concentration and minimal bactericidal concentration of GO, Ag, and GO:Ag

GO (mg/L)		5	2.5	1.25	0.625	0.3125	0.156
*S. aureus*	MIC	+	+	+	+	+	+
	MBC	+	+	+	+	+	+
*P. aeruginosa*	MIC	+	+	+	+	+	+
	MBC	+	+	+	+	+	+
Ag (mg/L)		25	12.5	6.25	3.125	1.56	0.78
*S. aureus*	MIC	-	-	-	-	+	+
	MBC	-	-	+	+	+	+
*P. aeruginosa*	MIC	-	-	-	-	+	+
	MBC	-	+	+	+	+	+
GO:Ag (mg/L)		5:25	2.5:12.5	1.25:6.25	0.625: 3.125	0.3125: 1.56	0.156: 0.78
*S. aureus*	MIC	-	-	-	-	-	+
	MBC	-	-	+	+	+	+
*P. aeruginosa*	MIC	-	-	-	-	+	+
	MBC	-	+	+	+	+	+

+ visible growth of bacteria,—lack of growth; MIC and MBC were defined as the lowest concentration that inhibits bacterial growth

Detailed analysis of GO:Ag antibacterial properties with proposed mechanisms of action were provided in our previous paper (Lange et al. 2022).

## Discussion

GO provides a large platform to deliver a variety of molecules, drugs, tracers, and nanoparticles (Jung et al. 2014; Campbell et al. 2019). Its chemical modification abilities in combination with low cytotoxicity provide GO with broad opportunities for biological and medical applications. In many applications, GO has been chemically modified, usually covalently bonded to PEG, to supply active substances (Ghosh and Chatterjee 2020). In the present study, pure GO and Ag were used to obtain the GO:Ag complex using the sonication method. Ultrasonic technologies have been proven to be an effective method of coating various materials with antibacterial agents (Perelshtein et al. 2010). In the prepared GO:Ag complex, a narrow band corresponding to -OH groups was observed (Fig. 3), which indicates limited interaction with water molecules, helping to evenly distribute Ag and ensuring better contact with cells. Ag showed strong adhesion to the GO surface (Fig. 1), which reduced the aggregation of bare nanoparticles (Table 1).

**Fig. 17:**
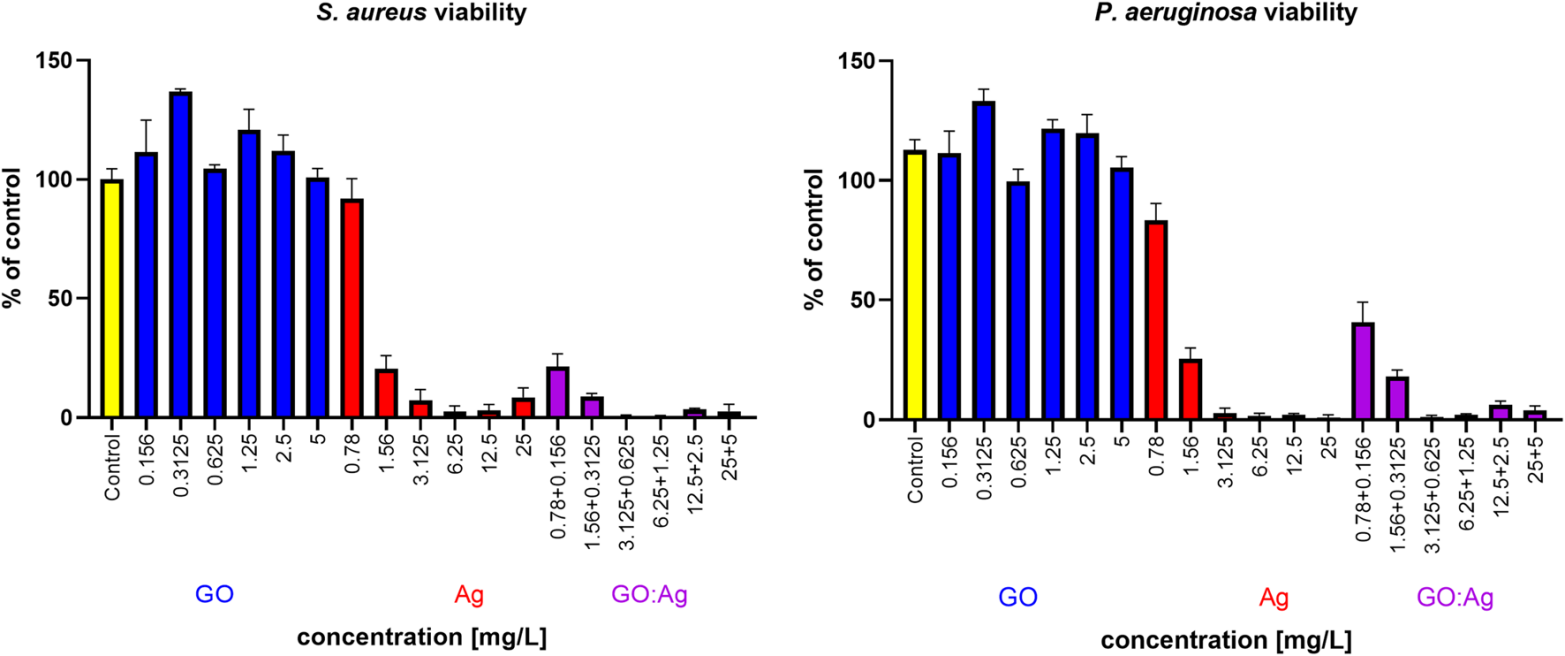
Bacteria viability determined by Presto Blue assay after 24 h of incubation with nanomaterials. All the suspensions were prepared accordingly to MIC method. Blue bars, GO; red bars, Ag; purple, GO:Ag

Ag, due to their antibacterial properties, have been used in the coatings of medical devices, orthopedic prostheses, textiles, cosmetics, and food storage and pharmaceutical products (Li et al. 2010). Every year, interest in the antibacterial properties of silver nanoparticles is growing due to the growing problem of antibiotic resistance. Despite the differences in antibacterial properties between different species of bacteria, this mechanism is quite universal, and its basis is mechanical damage of the bacterial cell and the secretion of Ag^+^ ions, followed by the disruption of the continuity of the cell membrane, inhibition of ATP synthesis, and overproduction of ROS (Zhang et al. 2016; Yin et al. 2020; Lange et al. 2022). Antibacterial Ag attached to the GO layer are more stable and well dispersed (Jaworski et al. 2018). In experiments on *S. aureus* and *P. aeruginosa*, a reduction in the number of bacteria was observed after incubation with Ag and the GO:Ag complex (Tables 2 and 3, Figs. 16 and 17). In our previous studies, it was observed that the bacteria were distributed over the entire surface of the graphene or GO (Kurantowicz et al. 2015; Lange et al. 2022). The attachment of bacteria to the surface of graphene oxide forces the contact of the bacterial cell with Ag, which increases the damage to bacterial cells.

Although the antibacterial effect of Ag and their complex with GO has been confirmed previously, there is a rising concern regarding safe application and toxicity of newly prepared complexes. The selection and performance of appropriate in vitro assays seems to be crucial to obtaining meaningful, reproducible, and scientifically reliable information on the cytotoxicity of nanostructures. In the case of the MTT assay, falsification of the results may be the consequence not only of MTT interference with the test factors or components of the culture medium (Ghasemi et al. 2021), which should always be excluded with cell-free samples, but also of the influence of the test substances on mitochondrial biogenesis, central points of mitochondrial regulation, and disturbances in Ca^2+^ homeostasis within the mitochondrial matrix, as a consequence of which the activity of mitochondrial dehydrogenases changes (Rai et al. 2018). It is recommended to perform several different cytotoxicity assays with standard microscopic controls in order to assess the actual cytotoxicity potential of the test agents. Therefore, the results of the MTT assay were compared with the results of the neutral red uptake and LDH leakage assays (Fig. 5). Decreased cell viability based on the neutral red uptake assay was demonstrated for GO:Ag complexes with concentrations of 1:5 mg/L and 5:5 mg/L for A549 cells and for Ag with a concentration of 5 mg/L for both cell lines (Fig. 5). The LDH leakage assay showed cytotoxicity, understood as damage to cell membranes, in A549 cells treated with GO at concentrations of 1 mg/L, 12.5 mg/L, and 25 mg/L; Ag at the concentration of 5 mg/L; and the GO:Ag complex at the concentration of 5:5 mg/L, while for the HFFF2 cell line, there was no evidence of damage to cell membranes based on the LDH assay. Studies on the A549 and Raw 264.7 cell lines have shown the possibility of creating water-permeable pores on cell membranes by transporting lipids from the protopore region to the GO surface, leading to a critical reduction in membrane density at characteristic interstitial sites, leakage of extracellular content, and ultimately, cell death (Duan et al. 2017). Ag attached to the GO platform can enhance the cytotoxic effect in A549 cells and induce apoptosis through mitochondrial damage and mitochondrial dysfunction, PINK1/Parkin-dependent mitophagy, and blocking autophagic flux. (Li et al. 2021) The internalization of Ag released from the GO surface occurs mainly in the phagocytic and endocytic pathways and can cause cell death as a result of lipid peroxidation and proteotoxic stress (Rohde et al. 2021). Moreover, in A549 cells, Ag are biotransformed into Ag complexed with thiol-containing proteins, which reduces the level of cellular metabolism and affects the blockade of the cell cycle (Bobyk et al. 2021). Depending on the time and dose of GO exposure, human lung cells may show different cellular responses (Mukherjee et al. 2020). Brief exposure to high doses results in cellular stress through mitochondrial dysfunction, with no evidence of cell death. In the case of chronic exposure to low doses, proapoptotic genes are upregulated and antiapoptotic genes are downregulated, and consequently resulting in sensitization to apoptosis induction in cells.

Interestingly, the ROS release assay demonstrated the induction of constant, dose-independent oxidative stress in the HFFF2 cell line, while the absence of this effect was demonstrated for the A549 line (Fig. 7). Studies on both normal (Shaheen et al. 2017; Ali et al. 2018) and neoplastic cell lines (Luna et al. 2016; Ali et al. 2018) show the effect of the GO:Ag complex on the increased release of ROS by cells. It is classically argued that increased ROS levels contribute to cell death by damaging macromolecules such as DNA, RNA, proteins, and lipids. However, in properly functioning cells, the physiological ROS level is a constant and indispensable component of many signaling pathways. Moreover, ROS can enhance the phenomenon of cell adaptation to new stimuli accompanying the change of the environment after administration of the test agents (Powers et al. 2010; Miller et al. 2019). After the ROS-mediated adaptation process, the cell can counteract the stress induced by changing environmental conditions, but as a result of a stimulus with an intensity exceeding the cell's adaptive capacity, the cell is directed to one of the pathways of cell death.

The results of the BrdU test initially confirmed the adaptation of the cells after treatment with the test agents. A decrease in the proliferation of cells treated for 24 h with Ag from a concentration of 3 mg/L and 4 mg/L was demonstrated for HFFF2 and A549 cells, respectively, and for GO:Ag complexes at concentrations of 1:5 mg/L and 5:5 mg/L for both lines (Fig. 6). Surprisingly, cell proliferation after 48 h of treatment was normalized and restored to the control level. The exception was the GO:Ag complex at the concentration of 5:5 mg/L, which contributed to the decrease in cell proliferation of both lines. The stimulating effect of GO, which appears only after cell adaptation and prolonged treatment, is confirmed by studies with the use of keratinocytes (Salesa and Serrano-Aroca 2021). However, this effect was observed only at low concentrations of GO (0.005 µg/mL and 0.01 µg/mL), similar to the study using graphene oxide quantum dots (GOQDs) with concentrations of 0.1 µg/mL and 1 µg/mL, which promoted the proliferation and osteogenic differentiation of bone marrow stromal cells by activating the Wnt/β-catenin signaling pathway (Xu et al. 2022). Enhanced proliferation was also observed in vascular smooth muscle cells by increasing the expression of Ki67, commonly considered a marker of cell proliferation (Ren et al. 2020). The coating of titanium with GO loaded with aspirin was demonstrated to increase the survival, proliferation, and osteogenic differentiation of MC3T3-E1 cells (Ren et al. 2018). Similarly, the incorporation of GO into the structure of biodegradable polyester matrices has a positive effect on the proliferation of preosteoblasts (Fu et al. 2017) and neural stem cells (Fu et al. 2019; Qi et al. 2020).

As the generation of oxidative stress in the cell can lead to cell death, the activity of caspase-3/7, which is an early indicator of apoptosis, was assessed using a novel probe. Caspase-3/7 activity was not detected by the fluorometric assay method in this study (Fig. 8). A weak signal of caspase-3/7 activity was observed in the confocal microscopy slides only for HFFF2 cells treated with 5 mg/L Ag (Figs. 9–11). Moreover, contraction of the cytoskeleton of HFFF2 cells treated with Ag 5 mg/L and GO:Ag 1:5 mg/L complex was demonstrated (Fig. 10). It has been shown previously that the association of GO with cell death may be size-dependent(Strojny et al. 2020). Subnanometric- and nanometric- sized GOs are presumed to induce cell death by autophagy via the PLCβ3/IP3/Ca^2+/^JNK signaling axis, while this effect was not observed for micrometric GO (> 1000 nm) and GO quantum dots (GOQDs) (Lim et al. 2016). A potential model of cell death induced by the GO:Ag complex assumes the maximization of contact with the cell membrane through GO sheets, thus allowing the cell to take up the complex via endocytosis (Luna et al. 2016) In view of the possibility of uptake of GO:Ag by cells, the possibility of biodegradation of GO sheets implies the problem of cytotoxicity of the decay products of the complex. However, the decay products of GO do not have a cytotoxic effect or degrade genetic material, as shown in a human lung cell model (Mukherjee et al. 2018).

The phenomenon of HFFF2 cell contraction (Figs. 10 and 11) can be explained by biomechanical analyses that prove the effect of GO on increasing cell stiffness and increasing the actin-actin distance, and then the detachment of actin subunits from each other in actin fibers, while not inducing cell death (Ghorbani et al. 2021). The influence of Ag is also important, as they may affect the loss of β-actin and F-actin, which are important components of the cytoskeleton (Xu et al. 2013). Ag also have the ability not only to bind actin and tubulin, but also to cause conformational changes in the above-mentioned proteins (Wen et al. 2013).

Microscopic analysis after live imaging showed no significant effect on cell morphology and density after 24 h and even 7 days of treatment (Fig. 12). Interestingly, when cells were maintained on GO and GO:Ag nanolayers (Figs. 13–15), a significant increase in the cell density of both cell lines was observed, while cells grown on Ag-coated surfaces showed increased density for A549 and decreased density for HFFF2 cells. The results indicate that GO:Ag nanolayers can be considered as a biocompatible cell scaffold and nanothick growth substrate with multidirectional action dedicated to many therapeutic approaches in tissue and biomaterials engineering. For example, GO nanolayers can be used in the process of muscle tissue regeneration by initiating the differentiation of muscle cells and activating proangiogenic signaling pathways (Zielińska-Górska et al. 2020; Wierzbicki et al. 2020). Moreover, we previously demonstrated that GO scaffolds combined with supplementation of chicken embryo liver extract can reduce the invasiveness of liver cancer cells by reducing cell proliferation, altering the expression of genes encoding protooncogenes, and entering the resting phase (Sosnowska et al. 2021).

GO:Ag nanolayers may constitute a cellular scaffold in their own right, but the GO:Ag complex can also be incorporated into the structure of well-known cellular scaffolds, improving their mechanical properties and eliciting the desired cellular response, thereby increasing the therapeutic effect. The hydrophilic nature of GO promotes cell adhesion by improving the wettability of the growth surface, enabling the derivation of advanced cell scaffolds based on a hydrophobic matrix, including polycaprolactone (PCL), polylactide-co-glycolide (PLGA), and polylactic acid (PLA). PLA which do not exhibit bactericidal properties, can be effectively used as carriers of the GO:Ag complex, thus constituting cytocompatible coatings with antibacterial properties (Liu et al. 2017; Bakhsheshi-Rad et al. 2020).

Additionally, providing a platform for the potentially cytotoxic nanoparticles, including silver nanoparticles, abolishes their cytotoxic effect, as shown in our recent research using human fibroblasts, human umbilical vein endothelial cells (HUVECs), the chorioallantoic membrane (CAM) (Wierzbicki et al. 2019), and the EpiDerm™ Skin irritation model (Zielińska-Górska et al. 2022). The above-mentioned reports support the need for detailed toxicity studies with sophisticated models aimed at assessing the long-term effect of various doses of the GO:Ag complex as well as individual components.

## Conclusion

The results confirmed the high cytocompatibility of GO, Ag, and the GO:Ag complex at selected concentrations. Direct studies of the cell cultures provide an insight into the molecular response of the cells, but further studies on the more complex models are necessary to completely confirm the biocompatibility of the newly presented material and promote its further application. We demonstrated that the GO:Ag complex acquires new features in comparison to the bare compounds. The essence of the GO:Ag complex formation is the elimination of the cytotoxic Ag effect, while maintaining their positive properties, including antibacterial properties, which can be provided by the deposition of Ag on the GO platform and the controlled release of Ag from the composite. The results combined with our previous findings confirm the possibility of employing the GO:Ag complex as a safe, antimicrobial agent, benefiting the environment and society by restricting antibiotic usage.

## Data Availability

The datasets used and analyzed during our study are available from the corresponding author on reasonable request.

## Abbreviations

Ag: Silver nanoparticles
ATCC: American Type Culture Collection (ATCC)
BrdU: 5-Bromo-2'-deoxyuridine
DAPI: 4′,6-Diamidino-2-phenylindole dihydrochloride
DCF-DA: Dichlorofluorescin diacetate
DMEM: Dulbecco’s Modified Eagle Medium
EDS: Energy-dispersive X-ray spectroscopy
FT-IR: Fourier-transformed infrared spectroscopy
GO: Graphene oxide
LDH: Lactate dehydrogenase
MBC: Minimal bactericidal concentration
MIC: Minimal inhibitory concentration
MFI: Mean fluorescence intensity
MGG: May–Grünwald and Giemsa staining
MTT: 3-(4,5-Dimethylthiazol-2-yl)-2,5-diphenyltetrazolium bromide
NR: Neutral red
PBS: Phosphate buffered saline
PLA: Polylactic acid
ROS: Reactive oxygen species
SEM: Scanning electron microscopy
TEM: Transmission electron microscopy

## References

[CR1] Ali D, Alarifi S, Alkahtani S, Almeer RS (2018) Silver-doped graphene oxide nanocomposite triggers cytotoxicity and apoptosis in human hepatic normal and carcinoma cells. Int J Nanomed 13:5685–5699. 10.2147/IJN.S165448 10.2147/IJN.S165448 PMC6161714 30288041

[CR2] Azizi-Lalabadi M, Hashemi H, Feng J, Jafari SM (2020) Carbon nanomaterials against pathogens; the antimicrobial activity of carbon nanotubes, graphene/graphene oxide, fullerenes, and their nanocomposites. Adv Colloid Interface Sci 284:102250. 10.1016/j.cis.2020.102250 32966964 10.1016/j.cis.2020.102250

[CR3] Bacakova L, Pajorova J, Tomkova M et al (2020) Applications of nanocellulose/nanocarbon composites: Focus on biotechnology and medicine. Nanomaterials 10:1–32. 10.3390/nano10020196 10.3390/nano10020196 PMC7074939 31979245

[CR4] Bakhsheshi-Rad HR, Ismail AF, Aziz M et al (2020) Co-incorporation of graphene oxide/silver nanoparticle into poly-L-lactic acid fibrous: A route toward the development of cytocompatible and antibacterial coating layer on magnesium implants. Mater Sci Eng C 111:110812. 10.1016/j.msec.2020.110812 10.1016/j.msec.2020.110812 32279830

[CR5] Basu J, Basu JK, Bhattacharyya TK (2010) The evolution of graphene-based electronic devices. Int J Smart Nano Mater 1:201–223. 10.1080/19475411.2010.510856

[CR6] Bhakya S, Muthukrishnan S, Sukumaran M, Grijalva M, Cumbal L, Franklin Benjamin JH, Senthil Kumar T, Rao MV (2016) Antimicrobial, antioxidant and anticancer activity of biogenic silver nanoparticles – an experimental report. RSC Adv 6:81436–81446. 10.1039/C6RA17569D

[CR7] Bobyk L, Tarantini A, Beal D et al (2021) Toxicity and chemical transformation of silver nanoparticles in A549 lung cells: Dose-rate-dependent genotoxic impact. Environ Sci Nano 8:806–821. 10.1039/d0en00533a

[CR8] Bold BE, Urnukhsaikhan E, Mishig-Ochir T (2022) Biosynthesis of silver nanoparticles with antibacterial, antioxidant, anti-inflammatory properties and their burn wound healing efficacy. Front Chem 10:1–13. 10.3389/fchem.2022.972534 10.3389/fchem.2022.972534 PMC9441807 36072703

[CR9] Bruna T, Maldonado-Bravo F, Jara P, Caro N (2021) Silver Nanoparticles and Their Antibacterial Applications. Int J Mol Sci 22(13):7202. 10.3390/ijms22137202 34281254 10.3390/ijms22137202 PMC8268496

[CR10] Campbell E, Hasan MT, Pho C et al (2019) Graphene Oxide as a Multifunctional Platform for Intracellular Delivery, Imaging, and Cancer Sensing. Sci Rep 9:416. 10.1038/s41598-018-36617-4 30674914 10.1038/s41598-018-36617-4 PMC6344482

[CR11] Duan G, Zhang Y, Luan B, et al (2017) Graphene-Induced Pore Formation on Cell Membranes. Sci Rep 7. 10.1038/srep42767 10.1038/srep42767 PMC5317030 28218295

[CR12] Fu C, Bai H, Hu Q et al (2017) Enhanced proliferation and osteogenic differentiation of MC3T3-E1 pre-osteoblasts on graphene oxide-impregnated PLGA-gelatin nanocomposite fibrous membranes. RSC Adv 7:8886–8897. 10.1039/c6ra26020a

[CR13] Fu C, Pan S, Ma Y et al (2019) Effect of electrical stimulation combined with graphene-oxide-based membranes on neural stem cell proliferation and differentiation. Artif Cells, Nanomed Biotechnol 47:1867–1876. 10.1080/21691401.2019.1613422 31076002 10.1080/21691401.2019.1613422

[CR14] Gao H, Hu G, Liu H (2019) Preparation of a Highly Stable Dispersion of Graphene in Water with the Aid of Graphene Oxide. Ind Eng Chem Res 58:17842–17849. 10.1021/acs.iecr.9b03771

[CR15] Garavand F, Cacciotti I, Vahedikia N, et al (2020) A comprehensive review on the nanocomposites loaded with chitosan nanoparticles for food packaging. Crit Rev Food Sci Nutr 1–34. 10.1080/10408398.2020.1843133 10.1080/10408398.2020.1843133 33153290

[CR16] Geim AK, Novoselov KS (2007) The rise of graphene. Nat Mater 6:183–191. 10.1038/nmat1849 17330084 10.1038/nmat1849

[CR17] Ghasemi M, Turnbull T, Sebastian S, Kempson I (2021) The mtt assay: Utility, limitations, pitfalls, and interpretation in bulk and single-cell analysis. Int J Mol Sci 22. 10.3390/ijms222312827 10.3390/ijms222312827 PMC8657538 34884632

[CR18] Ghorbani M, Soleymani H, Hashemzadeh H et al (2021) Microfluidic investigation of the effect of graphene oxide on mechanical properties of cell and actin cytoskeleton networks: experimental and theoretical approaches. Sci Rep 11:1–13. 10.1038/s41598-021-95624-0 34376720 10.1038/s41598-021-95624-0 PMC8355332

[CR19] Ghosh S, Chatterjee K (2020) Poly(Ethylene Glycol) Functionalized Graphene Oxide in Tissue Engineering: A Review on Recent Advances. Int J Nanomed 15:5991–6006. 10.2147/IJN.S249717 10.2147/IJN.S249717 PMC7656781 33192060

[CR20] He K, Zeng Z, Chen A et al (2018) Advancement of Ag–Graphene Based Nanocomposites: An Overview of Synthesis and Its Applications. Small 14:1–13. 10.1002/smll.201800871 10.1002/smll.201800871 29952105

[CR21] Jaworski S, Wierzbicki M, Sawosz E et al (2018) Graphene Oxide-Based Nanocomposites Decorated with Silver Nanoparticles as an Antibacterial Agent. Nanoscale Res Lett 13:116. 10.1186/s11671-018-2533-2 29687296 10.1186/s11671-018-2533-2 PMC5913058

[CR22] Jaworski S, Strojny-Cieślak B, Wierzbicki M, et al (2021) Comparison of the toxicity of pristine graphene and graphene oxide, using four biological models. Materials (Basel) 14. 10.3390/ma14154250 10.3390/ma14154250 PMC8348526 34361444

[CR23] Jung HS, Kong WH, Sung DK et al (2014) Nanographene Oxide-Hyaluronic Acid Conjugate for Photothermal Ablation Therapy of Skin Cancer. ACS Nano 8:260–268. 10.1021/nn405383a 24383990 10.1021/nn405383a

[CR24] Kurantowicz N, Sawosz E, Jaworski S et al (2015) Interaction of graphene family materials with Listeria monocytogenes and Salmonella enterica. Nanoscale Res Lett 10:23. 10.1186/s11671-015-0749-y 25685114 10.1186/s11671-015-0749-y PMC4312314

[CR25] Lange A, Sawosz E, Wierzbicki M et al (2022) Nanocomposites of Graphene Oxide—Silver Nanoparticles for Enhanced Antibacterial Activity: Mechanism of Action and Medical Textiles Coating 2022. Mater 15:3122. 10.3390/MA15093122 10.3390/ma15093122 PMC9100992 35591457

[CR26] Li W-R, Xie X-B, Shi Q-S et al (2010) Antibacterial activity and mechanism of silver nanoparticles on Escherichia coli. Appl Microbiol Biotechnol 85:1115–1122. 10.1007/s00253-009-2159-5 19669753 10.1007/s00253-009-2159-5

[CR27] Li J, Chang X, Shang M et al (2021) Mitophagy–lysosomal pathway is involved in silver nanoparticle-induced apoptosis in A549 cells. Ecotoxicol Environ Saf 208:111463. 10.1016/j.ecoenv.2020.111463 33130480 10.1016/j.ecoenv.2020.111463

[CR28] Liao C, Li Y, Tjong SC (2018) Graphene nanomaterials: Synthesis, biocompatibility, and cytotoxicity. Int J Mol Sci 19. 10.3390/ijms19113564 10.3390/ijms19113564 PMC6274822 30424535

[CR29] Lim MH, Jeung IC, Jeong J et al (2016) Graphene oxide induces apoptotic cell death in endothelial cells by activating autophagy via calcium-dependent phosphorylation of c-Jun N-terminal kinases. Acta Biomater 46:191–203. 10.1016/j.actbio.2016.09.018 27640918 10.1016/j.actbio.2016.09.018

[CR30] Liu C, Shen J, Yeung KWK, Tjong SC (2017) Development and Antibacterial Performance of Novel Polylactic Acid-Graphene Oxide-Silver Nanoparticle Hybrid Nanocomposite Mats Prepared by Electrospinning. ACS Biomater Sci Eng 3:471–486. 10.1021/acsbiomaterials.6b00766 33465942 10.1021/acsbiomaterials.6b00766

[CR31] Lorca-Ponce J, Urzúa E, Ávila-Salas F, Ramírez AM, Ahumada M (2023) Silver nanoparticle’s size and morphology relationship with their electrocatalysis and detection properties. Appl Surf Sci 617:156584. 10.1016/j.apsusc.2023.156584

[CR32] Luna LAV, Moraes ACM, Consonni SR et al (2016) Comparative in vitro toxicity of a graphene oxide-silver nanocomposite and the pristine counterparts toward macrophages. J Nanobiotechnol 14:12. 10.1186/s12951-016-0165-1 10.1186/s12951-016-0165-1 PMC4765018 26912341

[CR33] Marcano DC, Kosynkin DV, Berlin JM et al (2010) Improved Synthesis of Graphene Oxide. ACS Nano 4:4806–4814. 10.1021/nn1006368 20731455 10.1021/nn1006368

[CR34] Mariadoss AVA, Saravanakumar K, Sathiyaseelan A, Wang MH (2020) Preparation, characterization and anti-cancer activity of graphene oxide–silver nanocomposite. J Photochem Photobiol B Biol 210:111984. 10.1016/j.jphotobiol.2020.111984 10.1016/j.jphotobiol.2020.111984 32771914

[CR35] Miller IP, Pavlović I, Poljšak B, et al (2019) Beneficial role of ROS in cell survival: Moderate increases in H2 O2 production induced by hepatocyte isolation mediate stress adaptation and enhanced survival. Antioxidants 8. 10.3390/antiox8100434 10.3390/antiox8100434 PMC6826461 31581418

[CR36] Mouhat F, Coudert FX, Bocquet ML (2020) Structure and chemistry of graphene oxide in liquid water from first principles. Nat Commun 11:1–9. 10.1038/s41467-020-15381-y 32218448 10.1038/s41467-020-15381-y PMC7099009

[CR37] Mukherjee SP, Gliga AR, Lazzaretto B et al (2018) Graphene oxide is degraded by neutrophils and the degradation products are non-genotoxic. Nanoscale 10:1180–1188. 10.1039/c7nr03552g 29271441 10.1039/c7nr03552g

[CR38] Mukherjee SP, Gupta G, Klöditz K, et al (2020) Next-Generation Sequencing Reveals Differential Responses to Acute versus Long-Term Exposures to Graphene Oxide in Human Lung Cells. Small 16. 10.1002/smll.201907686 10.1002/smll.201907686 32227449

[CR39] Parnianchi F, Nazari M, Maleki J, Mohebi M (2018) Combination of graphene and graphene oxide with metal and metal oxide nanoparticles in fabrication of electrochemical enzymatic biosensors. Int Nano Lett 8:229–239

[CR40] Perelshtein I, Applerot G, Perkas N et al (2010) Ultrasound radiation as a ‘throwing stones’ technique for the production of antibacterial nanocomposite textiles. ACS Appl Mater Interfaces 2:1999–2004. 10.1021/am100291w 20614915 10.1021/am100291w

[CR41] Powers SK, Duarte J, Kavazis AN, Talbert EE (2010) Reactive oxygen species are signalling molecules for skeletal muscle adaptation. Exp Physiol 95:1–9 19880534 10.1113/expphysiol.2009.050526 PMC2906150

[CR42] Qi Z, Chen X, Guo W, et al (2020) Theanine-Modified Graphene Oxide Composite Films for Neural Stem Cells Proliferation and Differentiation. J Nanomater 2020. 10.1155/2020/3068173

[CR43] Rai Y, Pathak R, Kumari N et al (2018) Mitochondrial biogenesis and metabolic hyperactivation limits the application of MTT assay in the estimation of radiation induced growth inhibition. Sci Rep 8:1–15. 10.1038/s41598-018-19930-w 29367754 10.1038/s41598-018-19930-w PMC5784148

[CR44] Ren L, Pan S, Li H et al (2018) Effects of aspirin-loaded graphene oxide coating of a titanium surface on proliferation and osteogenic differentiation of MC3T3-E1 cells. Sci Rep 8:1–13. 10.1038/s41598-018-33353-7 30310118 10.1038/s41598-018-33353-7 PMC6181949

[CR45] Ren J, Braileanu G, Gorgojo P, et al (2020) On the biocompatibility of graphene oxide towards vascular smooth muscle cells. Nanotechnology 32. 10.1088/1361-6528/abc1a3 10.1088/1361-6528/abc1a3 33059341

[CR46] Rohde MM, Snyder CM, Sloop J et al (2021) The mechanism of cell death induced by silver nanoparticles is distinct from silver cations. Part Fibre Toxicol 18:37. 10.1186/s12989-021-00430-1 34649580 10.1186/s12989-021-00430-1 PMC8515661

[CR47] Salesa B, Serrano-Aroca Á (2021) Multi-Layer Graphene Oxide in Human Keratinocytes: Time-Dependent Cytotoxicity, Proliferation, and Gene Expression. Coatings 11:414. 10.3390/coatings11040414

[CR48] Shaheen F, Hammad Aziz M, Fakhar-e-Alam M et al (2017) An in vitro study of the photodynamic effectiveness of GO-AG nanocomposites against human breast cancer cells. Nanomaterials 7:401. 10.3390/nano7110401 29160836 10.3390/nano7110401 PMC5707618

[CR49] Sosnowska M, Kutwin M, Strojny B et al (2021) Graphene oxide nanofilm and chicken embryo extract decrease the invasiveness of HepG2 liver cancer cells. Cancer Nanotechnol 12:1–33. 10.1186/s12645-020-00073-533456622

[CR50] Strojny B, Jaworski S, Misiewicz-Krzemińska I et al (2020) Effect of graphene family materials on multiple myeloma and non-Hodgkin’s lymphoma cell lines. Materials (basel) 13:1–21. 10.3390/ma13153420 10.3390/ma13153420 PMC7436021 32756412

[CR51] Wang J, Ma F, Liang W, Sun M (2017) Electrical properties and applications of graphene, hexagonal boron nitride (h-BN), and graphene/h-BN heterostructures. Mater Today Phys 2:6–34. 10.1016/j.mtphys.2017.07.001

[CR52] Wen Y, Geitner NK, Chen R et al (2013) Binding of cytoskeletal proteins with silver nanoparticles. RSC Adv 3:22002–22007. 10.1039/c3ra43281e

[CR53] Wierzbicki M, Hotowy A, Kutwin M et al (2020) Graphene Oxide Scaffold Stimulates Differentiation and Proangiogenic Activities of Myogenic Progenitor Cells. Int J Mol Sci 21:4173. 10.3390/ijms21114173 32545308 10.3390/ijms21114173 PMC7311992

[CR54] Wierzbicki M, Jaworski S, Sawosz E, et al (2019) Graphene Oxide in a Composite with Silver Nanoparticles Reduces the Fibroblast and Endothelial Cell Cytotoxicity of an Antibacterial Nanoplatform. Nanoscale Res Lett 14. 10.1186/s11671-019-3166-9 10.1186/s11671-019-3166-9 PMC6787127 31602544

[CR55] Xu F, Piett C, Farkas S et al (2013) Silver nanoparticles (AgNPs) cause degeneration of cytoskeleton and disrupt synaptic machinery of cultured cortical neurons. Mol Brain 6:29. 10.1186/1756-6606-6-29 23782671 10.1186/1756-6606-6-29 PMC3695839

[CR56] Xu D, Wang C, Wu J et al (2022) Effects of Low-Concentration Graphene Oxide Quantum Dots on Improving the Proliferation and Differentiation Ability of Bone Marrow Mesenchymal Stem Cells through the Wnt/β-Catenin Signaling Pathway. ACS Omega 7:13546–13556. 10.1021/acsomega.1c06892 35559202 10.1021/acsomega.1c06892 PMC9088760

[CR57] Yildiz G, Bolton-Warberg M, Awaja F (2021) Graphene and graphene oxide for bio-sensing: General properties and the effects of graphene ripples. Acta Biomater 131:62–79. 10.1016/j.actbio.2021.06.047 34237423 10.1016/j.actbio.2021.06.047

[CR58] Yin IX, Zhang J, Zhao IS et al (2020) The Antibacterial Mechanism of Silver Nanoparticles and Its Application in Dentistry. Int J Nanomed 15:2555–2562. 10.2147/IJN.S246764 10.2147/IJN.S246764 PMC7174845 32368040

[CR59] Yu W, Sisi L, Haiyan Y, Jie L (2020) Progress in the functional modification of graphene/graphene oxide: a review. RSC Adv 10:15328–15345. 10.1039/D0RA01068E 35495479 10.1039/d0ra01068e PMC9052494

[CR60] Zhang X-F, Liu Z-G, Shen W, Gurunathan S (2016) Silver Nanoparticles: Synthesis, Characterization, Properties, Applications, and Therapeutic Approaches. Int J Mol Sci 17. 10.3390/ijms17091534 10.3390/ijms17091534 PMC5037809 27649147

[CR61] Zielińska-Górska M, Hotowy A, Wierzbicki M et al (2020) Graphene oxide nanofilm and the addition of l-glutamine can promote development of embryonic muscle cells. J Nanobiotechnology 18:1–17. 10.1186/s12951-020-00636-z 32414365 10.1186/s12951-020-00636-z PMC7229609

[CR62] Zielińska-Górska M, Sawosz E, Sosnowska M et al (2022) Molecular Biocompatibility of a Silver Nanoparticle Complex with Graphene Oxide to Human Skin in a 3D Epidermis In Vitro Model. Pharmaceutics 14:1398. 10.3390/pharmaceutics14071398 35890292 10.3390/pharmaceutics14071398 PMC9319156

